# The Zinc Finger Transcription Factor Fts2 Represses the Yeast-to-Filament Transition in the Dimorphic Yeast Yarrowia lipolytica

**DOI:** 10.1128/msphere.00450-22

**Published:** 2022-11-21

**Authors:** Jia-Wen Chen, Yi-Sheng Mao, Lv-Qiao Yan, Xiang-Dong Gao

**Affiliations:** a Hubei Key Laboratory of Cell Homeostasis, College of Life Sciences, Wuhan University, Wuhan, China; University of Georgia

**Keywords:** dimorphic transition, dimorphism, filamentation, hyphal growth, C_2_H_2_ zinc finger proteins

## Abstract

The yeast-to-filament transition is an important cellular response to environmental stimulations in dimorphic fungi. In addition to activators, there are repressors in the cells to prevent filament formation, which is important to keep the cells in the yeast form when filamentation is not necessary. However, very few repressors of filamentation are known so far. Here, we identify a novel repressor of filamentation in the dimorphic yeast Yarrowia lipolytica, Fts2, which is a C_2_H_2_-type zinc finger transcription factor. We show that *fts2*Δ cells exhibited increased filamentation under mild filament-inducing conditions and formed filaments under non-filament-inducing conditions. We also show that Fts2 interacts with YlSsn6, component of the Tup1-Ssn6 transcriptional corepressor, and Fts2-LexA represses a *lexAop-P_YlACT1_-lacZ* reporter in a Tup1-Ssn6-dependent manner, suggesting that Fts2 has transcriptional repressor activity and represses gene expression via Tup1-Ssn6. In addition, we show that Fts2 represses a large number of cell wall protein genes and transcription factor genes, some of which are implicated in the filamentation response. Interestingly, about two-thirds of Fts2-repressed genes are also repressed by Tup1-Ssn6, suggesting that Fts2 may repress the bulk of its target genes via Tup1-Ssn6. Lastly, we show that Fts2 expression is downregulated in response to alkaline pH and the relief of negative control by Fts2 facilitates the induction of filamentation by alkaline pH.

**IMPORTANCE** The repressors of filamentation are important negative regulators of the yeast-to-filament transition. However, except in Candida albicans, very few repressors of filamentation are known in dimorphic fungi. More importantly, how they repress filamentation is often not clear. In this paper, we report a novel repressor of filamentation in Y. lipolytica. Fts2 is not closely related in amino acid sequence to CaNrg1 and Rfg1, two major repressors of filamentation in C. albicans, yet it represses gene expression via the transcriptional corepressor Tup1-Ssn6, similar to CaNrg1 and Rfg1. Using transcriptome sequencing, we determined the whole set of genes regulated by Fts2 and identified the major targets of Fts2 repression, which provide clues to the mechanism by which Fts2 represses filamentation. Our results have important implications for understanding the negative control of the yeast-to-filament transition in dimorphic fungi.

## INTRODUCTION

Some fungal species can switch the morphology from the oval-shaped yeast form to filamentous forms in response to environmental stimulations (1–3). This yeast-to-filament transition (also called dimorphic transition or filamentation) is a stress response that helps the cells to expand into new environment. In some pathogenic fungi, including the human pathogen Candida albicans, the ability to switch between the yeast and filamentous forms is essential for virulence (1, 2). Therefore, the regulation of dimorphic transition has attracted a lot of attention. Many of these studies have been conducted in 2 yeast species, Saccharomyces cerevisiae and C. albicans, and extensive knowledge has been gained from these studies.

Yarrowia lipolytica is an industrial yeast utilized in the production of valuable metabolites including organic acids, sugar alcohols, and lipids (4, 5). Under the induction of poor carbon source, nitrogen starvation, and alkaline pH, Y. lipolytica cells can switch from oval-shaped yeast form to filamentous forms including rod-like elongated cells, pseudohyphae, and hyphae (6, 7). Previous studies have shown that filamentation in Y. lipolytica is positively regulated by evolutionarily conserved pathways such as the Ras/MAPK pathway and the Rim101 pH response pathway (8, 9), which also function similarly in S. cerevisiae and C. albicans (10–13). Filamentation is also positively regulated by the transcription factors Mhy1 and Hoy1 (14, 15). Mhy1 is a key regulator of filamentation in Y. lipolytica. Its deletion abolished filamentation whereas its overexpression caused strong filamentation (15, 16). Furthermore, proteins that regulate cytoskeletal organization including YlBem1, YlCla4, and YlRac1 are also required for filamentation (17–19).

In addition to positive regulators that promote filamentation under filament-inducing conditions, studies in C. albicans revealed the existence of negative regulators that repress filamentation under non-filament-inducing conditions. The transcription factors CaNrg1 and Rfg1, and the general transcriptional corepressor Tup1-Ssn6 play a major role in the repression of filamentation in C. albicans (20–24). Tup1-Ssn6 is an evolutionarily conserved corepressor complex responsible for the repression of hundreds of genes implicated in a variety of cellular functions in S. cerevisiae and C. albicans (25–28). Tup1 and Ssn6 do not bind DNA. They are brought to specific promoters by DNA-binding transcription factors to execute the repression of target genes. CaNrg1 and Rfg1 are thought to repress gene expression via Tup1-Ssn6 (21, 22, 28).

Previous studies have identified several negative regulators of filamentation in Y. lipolytica, including the Ras/PKA pathway, the high-osmolarity glycerol response (HOG) pathway, the TORC1-Sch9 pathway, and the transcription factors YlTec1 and Znc1 (29–33). Among these, the HOG pathway and the TORC1-Sch9 pathway function similarly in C. albicans. However, the Ras/PKA pathway and the transcription factor CaTec1 promote, but do not repress, filamentation in C. albicans (34, 35). Hence, there are similarities as well as differences between Y. lipolytica and C. albicans regarding the mechanisms for the control of filamentation.

In this study, we report the identification of a novel repressor of filamentation, Fts2, in the dimorphic yeast Y. lipolytica and address how Fts2 represses filamentation. We examined the transcriptional repressor activity of Fts2 and its relationship with the Tup1-Ssn6 corepressor. We also characterized the major target genes controlled by Fts2 using transcriptome sequencing. Lastly, we investigated whether Fts2 expression may change during the yeast-to-filament transition and, if so, its involvement in the induction of filamentation.

## RESULTS

### Identification of Fts2, a novel repressor of filamentation in Y. lipolytica.

Yali0E23518 is a putative transcription factor whose cellular function is not known in Y. lipolytica. In a mutant screen, we isolated 2 insertional loss-of-function mutants of *YALI0E23518*, both of which displayed increased filamentation (data not shown). Subsequent deletion of this gene in the wild-type strain generated an identical phenotype (see below). This finding suggests that Yali0E23518 represses filamentation. Based on the mutant phenotype, Yali0E23518 was named Fts2 (Filamentous 2).

**FIG 1:**
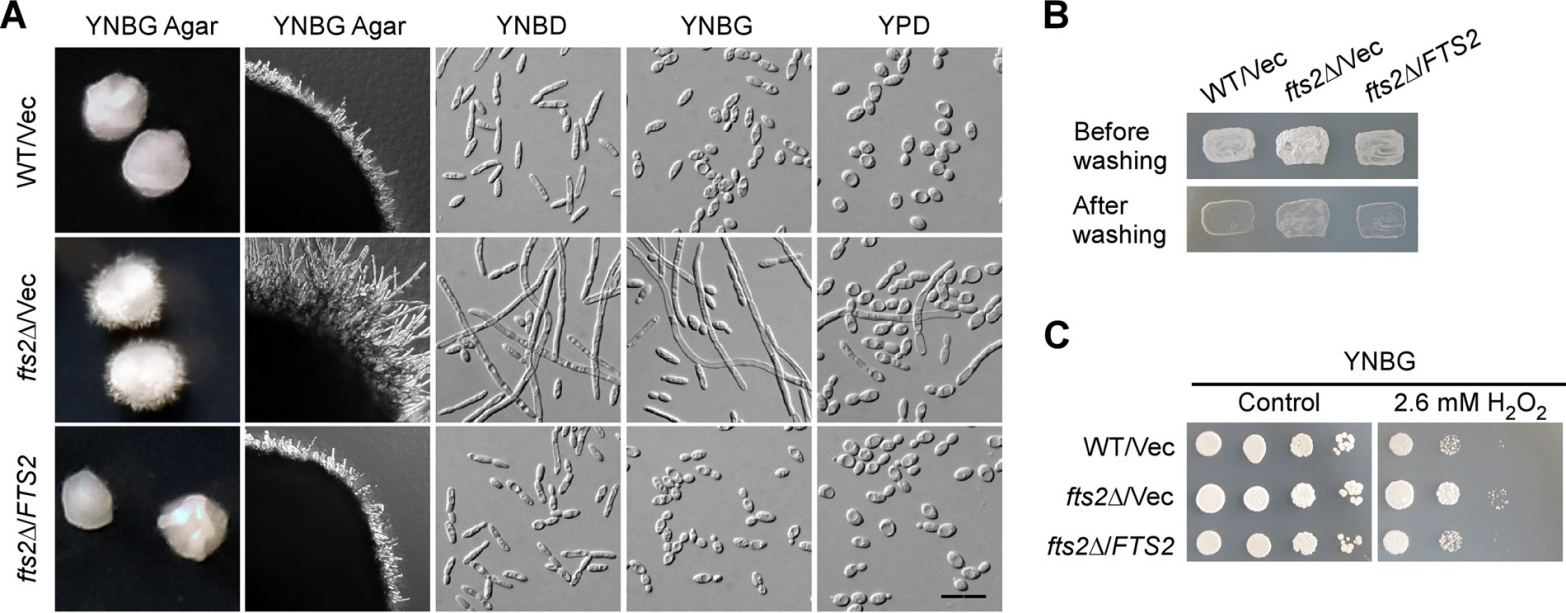
*fts2*Δ cells exhibited increased filamentation, stronger invasive growth, and increased resistance to peroxide. (A) *fts2*Δ cells exhibited increased filamentation compared with wild-type cells. Cells of wild-type strain (WT) carrying plasmid vector pINA445 (Vec) and *fts2*Δ strain carrying pINA445 or pINA445-FTS2 were grown at 30°C on YNBG agar for 2 days (left column), 1 day (second column), and in liquid YNBG (glycerol), YNBD (glucose), and YPD media for 16 h. (B) *fts2*Δ cells exhibited stronger invasive growth than wild-type cells. Cells as in (A) were grown on YNBG agar at 30°C for 2 days. (C) *fts2*Δ cells exhibited increased resistance to peroxide. Cells as in (A) were spotted at 1:10 serial dilution on YNBG agar with or without H_2_O_2_. Pictures were taken after grown at 30°C for 2 days. Bar, 20 μm.

We deleted the *FTS2* gene in the wild-type strain and examined the phenotypes of *fts2*Δ cells. *fts2*Δ cells grew normally as wild-type cells did. They did not exhibit any detectable defect in growth. When grown under mild filament-inducing conditions, such as on YNBG agar (a glycerol-containing synthetic medium), wild-type colonies were slightly wrinkled but not fluffy. In contrast, *fts2*Δ colonies were fluffy, covered with aerial filaments (Fig. 1A, left column). Microscopic examination of the microcolonies grown for a shorter time showed that *fts2*Δ colonies exhibited longer radial filaments than wild-type colonies (Fig. 1A, second column). Similarly, when grown in liquid YNBD medium, a glucose-containing medium that weakly stimulates filamentation, wild-type cells displayed an elongated, rod-like morphology but very few cells (~ 1%, *n *> 400) were longer than 30 μm. No long filaments were observed. In contrast, 64% of *fts2*Δ cells (*n *> 400) were longer than 30 μm, and long filaments were readily observed in *fts2*Δ cells (Fig. 1A, third column). These results indicate that *fts2*Δ cells exhibit increased filamentation compared with the wild-type cells under mild filament-inducing conditions, suggesting that Fts2 represses filamentation.

Interestingly, when grown under non-filament-inducing conditions, such as in liquid YNBG or YPD media, a significant fraction of *fts2*Δ cells were still elongated and formed filaments. In liquid YNBG medium, none of wild-type cells were longer than 20 μm. In contrast, 47% and 40% of *fts2*Δ cells (*n *> 400) were longer than 20 μm and 30 μm, respectively, and long filaments were readily observed (Fig. 1A, fourth column). In nutrient-rich YPD medium, none of wild-type cells were longer than 20 μm. In contrast, 19% and 10% of *fts2*Δ cells (*n *> 400) were longer than 20 μm and 30 μm, respectively, and long filaments were still present (Fig. 1A, right column). These results suggest that Fts2 also represses filamentation under non-filament-inducing conditions. Reintroduction of the *FTS2* gene into *fts2*Δ cells reduced filamentation to the level comparable to that of wild-type cells both on solid media and in liquid media, indicating that the loss of Fts2 function is responsible for increased filamentation in *fts2*Δ cells.

In addition to increased filamentation, *fts2*Δ cells also exhibited stronger invasive growth than wild-type cells (Fig. 1B). Moreover, *fts2*Δ cells exhibited increased resistance to peroxide (Fig. 1C), suggesting that Fts2 may also regulate the oxidative stress response.

Taken together, our results suggest that Fts2 is a novel repressor of filamentation. It also represses invasive growth and oxidative stress response.

### Fts2 is a C_2_H_2_ zinc finger transcription factor related to RfeC.

Fts2 is a protein of 301 amino acids featuring at the N-terminus two C_2_H_2_ zinc finger domains (a.a. 21 to 69), which are DNA-binding domains found in many transcription factors. Fts2 has no other recognizable domains except for a short QH-rich sequence (a.a. 148 to 192) in the central region.

**FIG 2:**
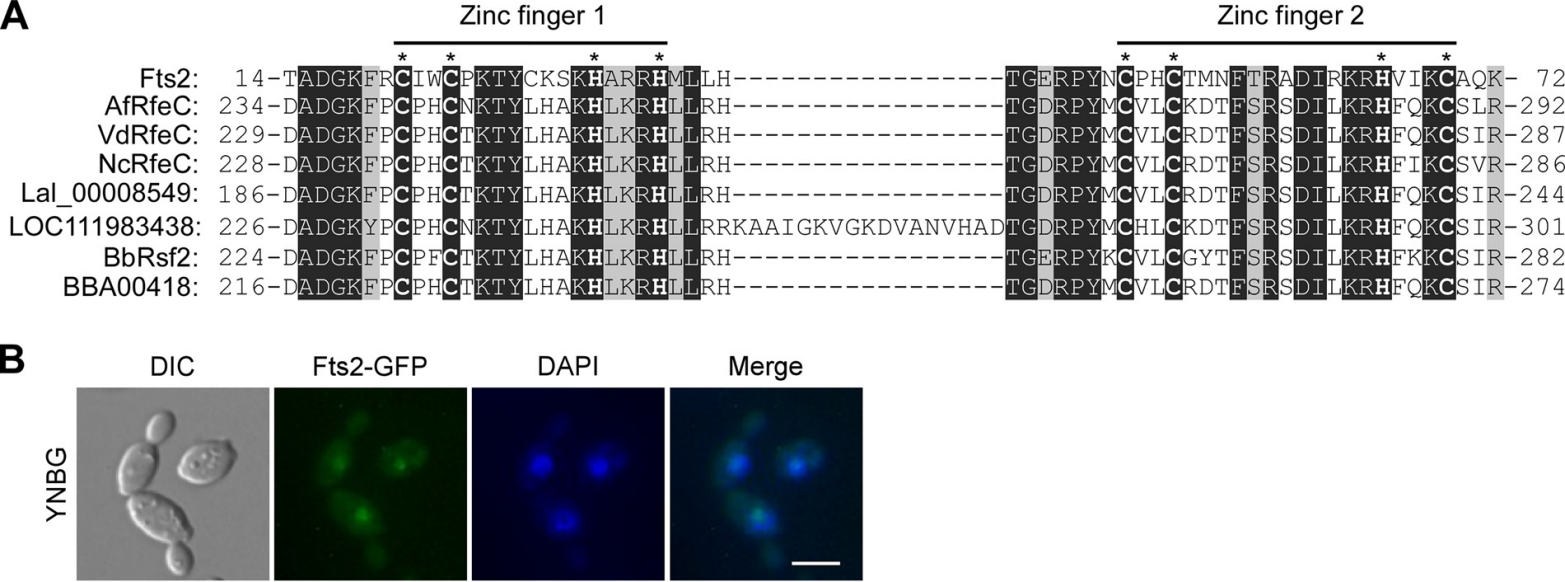
Fts2 is a C_2_H_2_ zinc finger transcription factor related to RfeC. (A) Fts2 is related to RfeC. Sequence alignment of Fts2 and RfeC proteins at the two C_2_H_2_ zinc finger domains plus the short flanking sequence. AfRfeC, Aspergillus flavus AF70 AFLA70_104g002430 (KOC14106.1); VdRfeC, Verticillium dahliae VdLs.17 VDAG_09001 (EGY18841.1); NcRfeC, Neurospora crassa OR74A, locus tag NCU05285 (EAA32820.1); Lal_00008549, plant Lupinus albus KAF1854605.1; LOC111983438, plant Quercus suber XP_023870868.1; BbRsf2, Beauveria bassiana D1-5 BBAD15_g11497 (KGQ03276.1); BBA00418, Beauveria bassiana ARSEF 2860 EJP70788.1. Identical and similar residues are highlighted in dark and gray shades, respectively. (B) Fts2-GFP localizes to the nucleus. Cells of wild-type strain carrying plasmid pYL14-FTS2 (expresses Fts2-GFP) were grown in YNBG medium. The nucleus was visualized by staining the cells with DAPI. Bar, 10 μm.

Fts2 does not share extensive amino acid sequence similarities to proteins in S. cerevisiae, C. albicans, and other yeasts. The closest homologs of Fts2 in S. cerevisiae are Mig2 and Mig1, whereas in C. albicans are Try5, Bcr1, and CaMig1. Fts2 shares 37% to 42% sequence identity to these proteins but the homologous region is limited to the 2 zinc finger domains. Fts2 shares a higher sequence identity (46% to 54%) to RfeC, the C_2_H_2_ zinc finger transcription factor widely present in filamentous fungi, and to the RfeC-like proteins in 2 plant species, Lupinus albus and Quercus suber (Fig. S1). However, the homologous region is still limited to the 2 zinc finger domains plus the flanking several amino acids (Fig. 2A). Outside this region, Fts2 does not share sequence similarity to these proteins. The cellular function of RfeC is not known except that Aspergillus terreus RfeC can promote *FLO11* expression when expressed in S. cerevisiae (36). Consistent with a predicted role as a transcription factor, GFP-tagged Fts2 localized to the nucleus (Fig. 2B). Thus, Fts2 is a novel RfeC-like C_2_H_2_ zinc finger transcription factor that controls filamentation.

10.1128/msphere.00450-22.1 FIG S1 Fts2 is related to RfeC C_2_H_2_ zinc finger transcription factors. Phylogenetic tree of Fts2 with related C_2_H_2_ zinc finger transcription factors. The neighbor-joining tree was constructed based on the full-length amino acid sequences of listed proteins using MEGA X. The scale bar represents 10 amino acid substitutions per site. ScMig1 and ScMig2, S. cerevisiae Mig1 and Mig2; CaMig1, C. albicans Mig1; CaTry5, C. albicans Try5; CaBcr1, C. albicans Bcr1; AnRfeC, Aspergillus nidulans AN5966 (XP_663570.1); AfRfeC, Aspergillus flavus AF70 AFLA70_104g002430 (KOC14106.1); VdRfeC, Verticillium dahliae VdLs.17 VDAG_09001 (EGY18841.1); NcRfeC, Neurospora crassa OR74A NCU05285 (EAA32820.1); Lal_00008549, plant *Lupinus albus* KAF1854605.1; LOC111983438, plant *Quercus suber* XP_023870868.1; BbRsf2, Beauveria bassiana D1-5 BBAD15_g11497 (KGQ03276.1); BBA00418, Beauveria bassiana ARSEF 2860 EJP70788.1. Download FIG S1, TIF file, 1.4 MB. Copyright © 2022 Chen et al. 2022 Chen et al. https://creativecommons.org/licenses/by/4.0/ This content is distributed under the terms of the Creative Commons Attribution 4.0 International license.

### Fts2 interacts with YlSsn6, component of the Tup1-Ssn6 general transcriptional corepressor, and Fts2-LexA represses a reporter gene in a Tup1-Ssn6-dependent manner.

**FIG 3:**
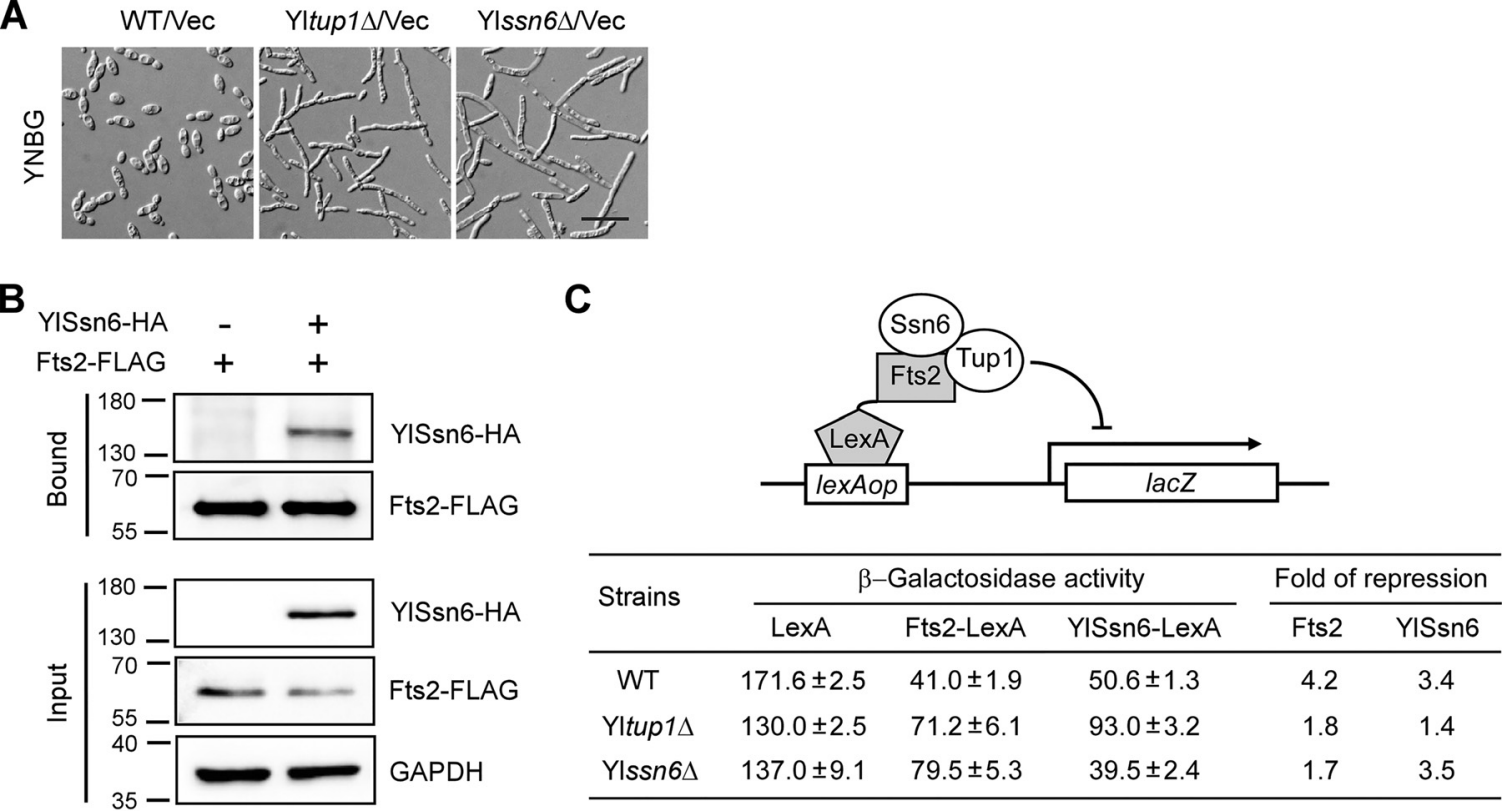
Fts2 interacts with YlSsn6 and Fts2-LexA represses a reporter gene in a Tup1-Ssn6-dependent manner. (A) Yl*tup1*Δ and Yl*ssn6*Δ cells formed filaments under non-filament-inducing condition. Cells of wild-type (WT), Yl*tup1*Δ, and Yl*ssn6*Δ strains carrying plasmid vector pINA445 (Vec) were grown in liquid YNBG medium at 30°C. Bar, 20 μm. (B) Fts2 interacts with YlSsn6 in co-immunoprecipitation assay. Cells of strain YLX524 (*FTS2-3FLAG*) carrying pINA445 vector or pINA445-YlSSN6-HA were grown in liquid YNBG (pH 4.0) medium. Cell lysates were subjected to immunoprecipitation with anti-FLAG affinity beads. YlSsn6-HA, Fts2-FLAG, and GAPDH were detected by immunoblotting with anti-FLAG, anti-HA, and anti-GAPDH antibodies, respectively. (C) Fts2-LexA represses *lexAop-P_YlACT1_-lacZ* expression in Y. lipolytica. Plasmids pINA443-P_YlTEF1_-lexA, pINA443-P_YlTEF1_-FTS2-lexA, and pINA443-P_YlTEF1_-YlSSN6-lexA that express the DNA-binding domain (a.a. 1 to 87) of LexA or Fts2-LexA and YlSsn6-LexA fusion proteins were transformed into wild-type, Yl*tup1*Δ, and Yl*ssn6*Δ strains carrying the reporter plasmid pINA445-lexAop-P_YlACT1_-*lacZ*. The reporter gene contains the *lacZ* gene under the control of four copies of the *lexA* operator and the Yl*ACT1* promoter. Cells were grown in liquid YNBG medium. Cell lysates were measured for β-galactosidase activity.

Tup1-Ssn6 is a general transcriptional corepressor in yeast. It is involved in the repression of hundreds of genes directed by specific transcription factors (25, 27, 28). In C. albicans, Tup1-Ssn6 represses filamentation (20, 27, 37). Similar to C. albicans Ca*tup1*Δ cells, Yl*tup1*Δ and Yl*ssn6*Δ cells also displayed increased filamentation (Fig. 3A), indicating that Tup1-Ssn6 also represses filamentation in Y. lipolytica. Interestingly, Fts2 interacted with YlSsn6 in co-immunoprecipitation assay in Y. lipolytica cells (Fig. 3B), suggesting that Fts2 may interact with the Tup1-Ssn6 corepressor.

To examine whether Fts2 has transcriptional repressor activity, we performed one-hybrid assay in Y. lipolytica cells, which tested the ability of a Fts2-LexA fusion (containing the DNA-binding domain of LexA, a.a. 1 to 87) to influence the expression of a *lexAop-P_YlACT1_-lacZ* reporter. Comparison of the expression levels of the reporter in cells expressing Fts2-LexA and LexA alone indicates that Fts2 caused 4.2-fold repression in wild-type cells (Fig. 3C). YlSsn6 caused 3.4-fold repression, comparable to that of Fts2. The repression by Fts2 was significantly reduced in Yl*tup1*Δ and Yl*ssn6*Δ cells (Fig. 3C), indicating that both YlTup1 and YlSsn6 are required for the repression by Fts2. This feature is very similar to that of CaNrg1 (25, 27, 28), a transcriptional repressor in C. albicans. Together, our results suggest that Fts2 represses gene expression via Tup1-Ssn6.

### Identification of Fts2-regulated genes by transcriptome sequencing.

**FIG 4:**
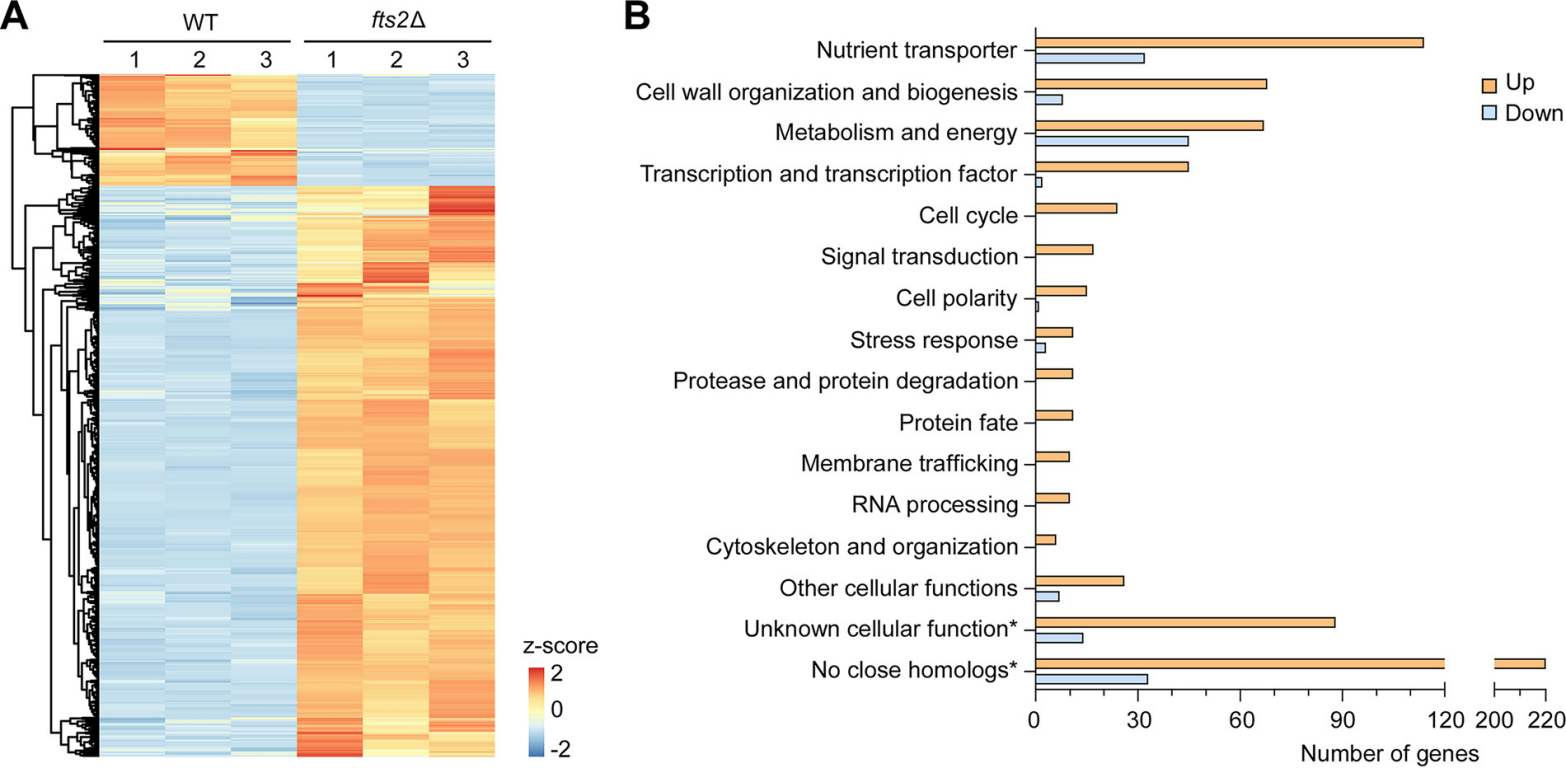
Fts2-regulated genes identified by transcriptome sequencing. (A) The heat map of the differentially expressed genes in *fts2*Δ cells compared with wild-type cells. (B) The numbers of upregulated and downregulated genes in functional categories. “Unknown cellular function” refers to genes that encode proteins similar to function-unknown proteins. *, in comparison with proteins in S. cerevisiae, C. albicans, or other fungi.

To understand how Fts2 represses filamentation, we wanted to identify the whole set of genes regulated by Fts2. To this end, we conducted RNA-Seq-based transcriptome sequencing in wild-type and *fts2*Δ cells grown in liquid YNBD medium. To determine whether the Fts2-regulated genes may also be controlled by Tup1-Ssn6, Yl*tup1*Δ cells and Yl*ssn6*Δ cells were also included in the analysis. In the RNA-Seq datasets, 6397 protein-coding genes (99.2%) in the genome (6448 protein-coding genes) were detected to be expressed in both wild-type and *fts2*Δ cells (Table S1, full data). Among these genes, a total of 888 genes displayed significant differential expression (≥2-fold, *P* < 0.05) in *fts2*Δ cells. Of these, 743 genes (83.7%) were upregulated whereas just 145 genes (16.3%) were downregulated (Fig. 4A and Table S1, upregulated and downregulated genes). A total of 202 genes displayed highly differential expression (≥5-fold, *P* < 0.05) in *fts2*Δ cells. Of these, 177 genes (87.6%) were highly upregulated, whereas only 25 genes (12.4%) were highly downregulated. In both cases, the number of upregulated genes greatly exceeds that of downregulated genes, suggesting that Fts2 functions mainly as a repressor in the control of gene expression.

10.1128/msphere.00450-22.2 TABLE S1 RNA-Seq data- *fts2*Δ vs WT. Download Table S1, XLSX file, 1.2 MB. Copyright © 2022 Chen et al. 2022 Chen et al. https://creativecommons.org/licenses/by/4.0/ This content is distributed under the terms of the Creative Commons Attribution 4.0 International license.

The upregulated genes in *fts2*Δ cells mainly encode nutrient transporters, cell wall proteins and enzymes involved in cell wall organization and biogenesis, metabolic enzymes, transcription factors, as well as proteins involved in cell cycle, signal transduction, cell polarity, and stress response (Fig. 4B and Table S2). In contrast, the downregulated genes mainly encode metabolic enzymes and nutrient transporters. A significant portion of the upregulated genes (41.5%) and the downregulated genes (32.5%) do not have a known cellular function. Some have homologs in S. cerevisiae, C. albicans, or other fungi but without known cellular functions. Many more do not have homologs in these species.

10.1128/msphere.00450-22.3 TABLE S2 Functional categories of the upregulated and downregulated genes in *fts2*Δ cells. Download Table S2, PDF file, 0.5 MB. Copyright © 2022 Chen et al. 2022 Chen et al. https://creativecommons.org/licenses/by/4.0/ This content is distributed under the terms of the Creative Commons Attribution 4.0 International license.

### Fts2 represses a large number of cell wall protein genes, including Yl*PHR1* and a set of adhesin-like genes.

**FIG 5:**
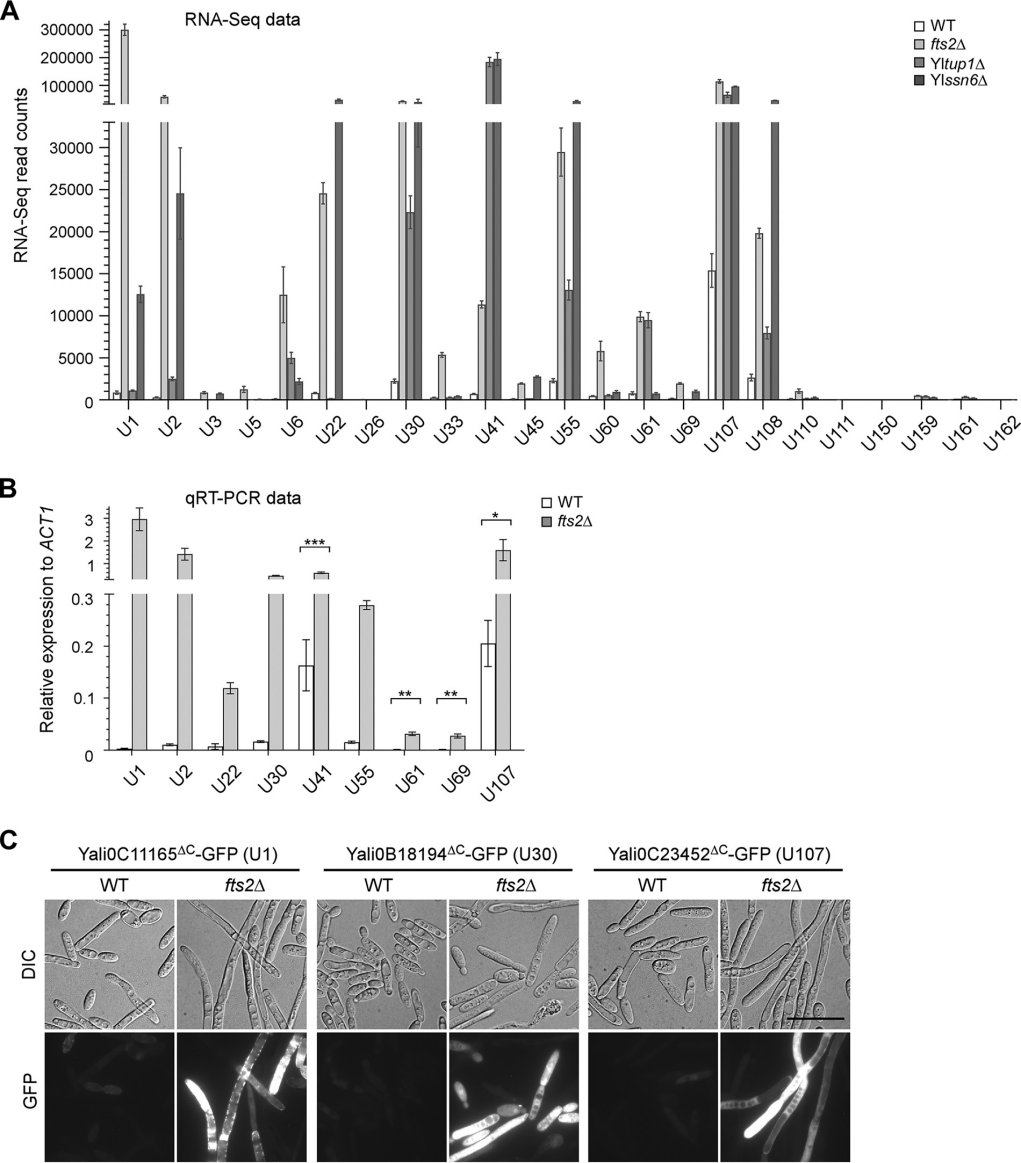
Fts2 represses a large number of cell wall protein genes. (A) The RNA-Seq read counts of the 23 highly Fts2-repressed (≥5-fold, *P* < 0.05) cell wall protein genes (U1-U162) in wild-type (WT), *fts2*Δ, Yl*tup1*Δ, and Yl*ssn6*Δ strains. (B) Validation of the RNA-Seq results by qRT-PCR. The transcript levels of U1, U2, U22, U30, U41, U55, U61, U69, and U107 in wild-type (WT) and *fts2*Δ strains grown in YNBD medium at 30°C was determined by qRT-PCR and normalized to Yl*ACT1*. Statistically significant differences are indicated by the asterisks (*, *P* < 0.05; **, *P* < 0.01; ***P < 0.001). (C) Fts2 represses three adhesin-like genes. Cells of wild-type (WT) and *fts2*Δ strains carrying pYL14-YALI0C11165^ΔC^, pYL14-YALI0B18194^ΔC^, and pYL14-YALI0C23452^ΔC^ were grown in YNBD medium and photographed for GFP fluorescence. Bar, 20 μm.

During the yeast-to-filament transition, the cell wall undergoes dramatic reorganization to support filamentous growth and modulate adherence properties (38). This process involves the upregulation and downregulation of some cell wall proteins. In the RNA-Seq data sets, we observed that a large number of cell wall protein genes were upregulated in *fts2*Δ cells. A total of 68 genes were significantly upregulated (≥2-fold, *P* < 0.05), whereas just 8 genes were significantly downregulated. Of these, 23 genes were highly upregulated (≥5-fold, *P* < 0.05), whereas only 1 gene was highly downregulated (Table 1). To evaluate the accuracy of the RNA-Seq data, 9 highly upregulated genes were examined for their mRNA levels by qRT-PCR. Consistent with the RNA-Seq data (Fig. 5A), all 9 genes were markedly upregulated at the transcription level in *fts2*Δ cells (Fig. 5B). We tagged the 3 highly upregulated adhesin-like genes *YALI0C11165* (U1), *YALI0B18194* (U30), and *YALI0C23452* (U107) at the 3′-terminus of ORF with GFP to allow the detection of these proteins in live cells (the short C-terminus containing the GPI modification site was deleted from each protein to avoid the removal of tagged GFP during GPI modification). *fts2*Δ cells carrying the 3 GFP-tagged genes all exhibited brighter GFP fluorescence than wild-type cells carrying the same construct (Fig. 5C), indicating that all 3 adhesin-like genes were upregulated at the protein level as well in *fts2*Δ cells.

**TABLE 1:** The upregulated and downregulated cell wall protein genes in *fts2*Δ cells identified by RNA-Seq^*a*^

Direction of regulation	Tup1-regulated only	Ssn6-regulated only	Tup1-Ssn6-coregulated	Non-Tup1-Ssn6 -regulated
Up High (≥5-fold) 23 genes	**E22286** (U61)	***C11165*** (U1), **D03740** (U5), ***DCW4*** (**F21857**, U45), *E19426* (U60)	***C15004*** (U2)^*b*^^,^^*c*^, **E22088** (U6)^*b*^^,^^*c*^, **B18194** (U30)^*b*^^,^^*c*^, ***YWP1*** (**E11517**, U41)^*b*^^,^^*c*^, ***PHR1*** (**D04851**, U55)^*b*^^,^^*c*^, ***C23452*** (U107)^*c*^, **E31108** (U108)^*c*^, **A08800** (U150)^*b*^^,^^*c*^, E20229 (U159), **E22440** (U161)^*b*^^,^^*c*^, **A15378** (U3)^*b*^^,^^*c*^^,^^*d*^, **F07535** (U22)^*b*^^,^^*c*^^,^^*d*^, **D09185** (U69)^*c*^^,^^*d*^	*F15653* (U26), *C08473* (U33), *B05654* (U110), *A09196* (U111), *D26257* (U162)
Low (≥2-fold, <5-fold) 45 genes	*C17875* (U218), *D17248* (U545), *DFG5* (F18722, U704) C13970 (U210)^*d*^, *D18381* (U439)^*d*^, *E08008* (U467)^*d*^, *E22550* (U493)^*d*^, *E25784* (U632)^*d*^	**F10549** (U306), ***C10923*** (U349), *E33363* (U418), *D06996* (U483), *CHS2* (*B16324*, U521), *CHS1* (*D03179*, U576), *B00132* (U620), *F21428* (U712)	*MSB2* (*D00627*, U186), **B00374** (U193)^*b*^^,^^*c*^, ***F19030*** (U202)^*b*^^,^^*c*^, ***E33891*** (U219)^*b*^^,^^*c*^, ***C14938*** (U244)^*c*^, ***CWP1*** (**E18788**, U261)^*b*^^,^^*c*^, *D27214* (U269), ***A21373*** (U298)^*b*^^,^^*c*^, D02024 (U334), ***F00990*** (U341)^*b*^, **E26125** (U381)^*b*^^,^^*c*^, *A18524* (U396), *F01925* (U398), *D10835* (U480), ***D24277*** (U486)^*b*^^,^^*c*^, *E30107* (U502), **C18293** (U584)^*b*^, ***F18282*** (U688)^*b*^^,^^*c*^, *D20680* (U721) **E01210** (U180)^*b*^^,^^*d*^, ***F22847*** (U217)^*b*^^,^^*d*^	A15796 (U230), *F10901* (U248), *E13860* (U302), *B18216* (U370), C08349 (U429), *TOS1* (A17919, 561), *A03597* (U615), *C14630* (U673)
DownHigh (≥5-fold) 1 gene		**E18766** (D12)		
Low (≥2-fold, <5-fold) 7 genes	*E03938* (D58), *C20779* (D135) *A20438* (D33)^*d*^, *D24101* (D68)^*d*^	*D01331* (D31)^*e*^	**D22957** (D73)^*b*^^,^^*e*^	*E28336* (D40)

a For simplicity, “*YALI0*” in the systematic name of each gene was omitted as “E22286” stands for “*YALI0E22286*”. Numbers in parentheses indicate each gene’s ranking in the full list of RNA-Seq data set. Genes encoding putative cell surface adhesins that share similarities to the known adhesins such as S. cerevisiae Flo11 and C. albicans Hyr1 or Hwp1 are underlined. Genes that displayed significant differential expression (≥2-fold, *P* < 0.05) in Yl*tup1*Δ or Yl*ssn6*Δ cells compared with wild-type cells are defined as YlTup1-regulated or YlSsn6-regulated. Genes that displayed highly differential expression (≥5-fold, *P *< 0.05) in Yl*tup1*Δ or Yl*ssn6*Δ cells are in bold.

b Genes that are highly differentially expressed in Yl*tup1*Δ cells.

c Genes that are highly differentially expressed in Yl*ssn6*Δ cells.

d The regulation on this gene by YlTup1 is opposite to the regulation by Fts2.

e The regulation on this gene by YlSsn6 is opposite to the regulation by Fts2.

Among the 68 upregulated cell wall protein genes, 9 genes encoding proteins similar to cell surface adhesins (*YALI0C11165*, *YALI0B8194*, and *YALI0C23452*), S. cerevisiae cell wall structural proteins Tir1/Tir3 (*YALI0E22088* and *YALI0F07535*) and Cwp1 (*YALI0E11517*, *YALI0E22286*, and *YALI0E31108*), and cell surface glycosidase (Yl*PHR1*) were not only highly upregulated (≥5-fold), but also exhibited very high RNA-Seq read counts (>9500) in *fts2*Δ cells (Table 1 and Table S1). Hence, these genes appear to be the major targets of Fts2 repression. Of these, *YALI0C11165* was upregulated by 338-fold, the highest among all genes. *YALI0E22088* and *YALI0F07535* were upregulated by 88.7-fold and 29.4-fold, respectively. Yl*PHR1* was upregulated by 12.8-fold. Among these genes, only Yl*PHR1* is known to be required for filamentation (9). The roles of other major target genes in filamentation are not clear. Some of them might play roles in filamentation (see Discussion).

Cell surface adhesins represented by the S. cerevisiae flocculin Flo11 and the C. albicans adhesins Hyr1 and Hwp1 are GPI-anchored cell surface glycoproteins that direct cell-cell adhesion or cell-surface adhesion (39, 40). *FLO11*, *HYR1*, and *HWP1* are filament-specific genes that are upregulated concomitant with the yeast-to-filament transition (41–43). We observed that a total of 18 adhesin-like genes were upregulated in *fts2*Δ cells (Table 1, underlined genes). Nine of them were highly upregulated (≥5-fold). These adhesin-like genes might be involved in modulating specific properties of the cell wall.

### Fts2 represses a large number of transcription factor genes, some of which promote filamentation.

Transcription factors play important roles in the control of gene expression. Our RNA-Seq analysis revealed that 41 transcription factor genes were significantly upregulated (≥2-fold, *P* < 0.05) in *fts2*Δ cells compared with wild-type cells. Six of these transcription factor genes were highly upregulated (≥5-fold, *P* < 0.05) (Table 2). In contrast, just 1 transcription factor gene was downregulated in *fts2*Δ cells.

**TABLE 2:** The upregulated and downregulated transcription factor genes in *fts2*Δ cells identified by RNA-Seq^*a*^

Direction of regulation	Tup1-regulated only	Ssn6-regulated only	Tup1-Ssn6-coregulated	Non-Tup1-Ssn6 -regulated
Up High (≥5-fold) 6 genes			***E14971*** (U37)^*b*^^,^^*c*^, *C09482* (U76), ***MHY1*** (**B21582**, U77)^*b*^^,^^*c*^, ***D10681*** (U114)^*b*^^,^^*c*^, ***BRG1*** (**E31757**, U127)^*b*^^,^^*c*^, **E34925** (U151)^*b*^^,^^*c*^	
Low (≥2-fold, <5-fold)35 genes	*C10010* (U289), *C03564* (U366), *YAP1* (*F03388*, U637), *PPR1* (*B09713*, U677), *C13178* (U706) *B11902* (U530)^*d*^	*GZF1* (*D20482*, U299), *F03135* (U402), *F03157* (U592) *F15543* (U273)^*e*^	**D14872** (U190)^*b*^^,^^*c*^, **B13354** (U199)^*b*^^,^^*c*^, ***AZF1*** (**A16841**, U203)^*b*^^,^^*c*^, ***PHD1*** (**B19602**, U208)^*b*^^,^^*c*^, **STP3** (**B05478**, U221)^*b*^, ***HOY1*** (**A18469**, U223)^*b*^, ***E15510*** (U262)^*b*^, ***B12716*** (U264)^*b*^, ***NRG1*** (**C12364**, U300)^*b*^^,^^*c*^, **D05041** (U320)^*b*^^,^^*c*^, ***WOR4*** (**F19822**, U394)^*b*^^,^^*c*^, ***MSN4*** (***C13750***, U403)^*b*^^,^^*c*^, *F25861* (U463), ***C01463*** (U454)^*b*^, *F16511* (U478), *F17468* (U482), **D04466** (U485)^*b*^, ***FTS1*** (**B04510**, U505)^*b*^, ***TEC1*** (**F15169**, U566)^*b*^, ***GZF2*** (**F17886**, U605)^*b*^, *C11858* (U606), *SFL1* (*D04785*, U727), ***C08327*** (U738)^*b*^^,^^*c*^	*B13200* (U537), *E11693* (U585)
Down High (≥5-fold)0 gene				
Low (≥2-fold, <5-fold)1 gene			***E17215*** (D99)*^b,d,e^*	

a For simplicity, “*YALI0*” in the systematic name of each gene was omitted as “E22286” stands for “*YALI0E22286*”. Numbers in parentheses indicate each gene’s ranking in the full list of RNA-Seq data set. Genes that displayed significant differential expression (≥2-fold, *P* < 0.05) in Yl*tup1*Δ or Yl*ssn6*Δ cells compared with wild-type cells are defined as YlTup1-regulated or YlSsn6-regulated. Genes that displayed highly differential expression (≥5-fold, *P *< 0.05) in Yl*tup1*Δ or Yl*ssn6*Δ cells are in bold.

b Genes that are highly differentially expressed in Yl*tup1*Δ cells.

c Genes that are highly differentially expressed in Yl*ssn6*Δ cells.

d The regulation on this gene by YlTup1 is opposite to the regulation by Fts2.

e The regulation on this gene by YlSsn6 is opposite to the regulation by Fts2.

**FIG 6:**
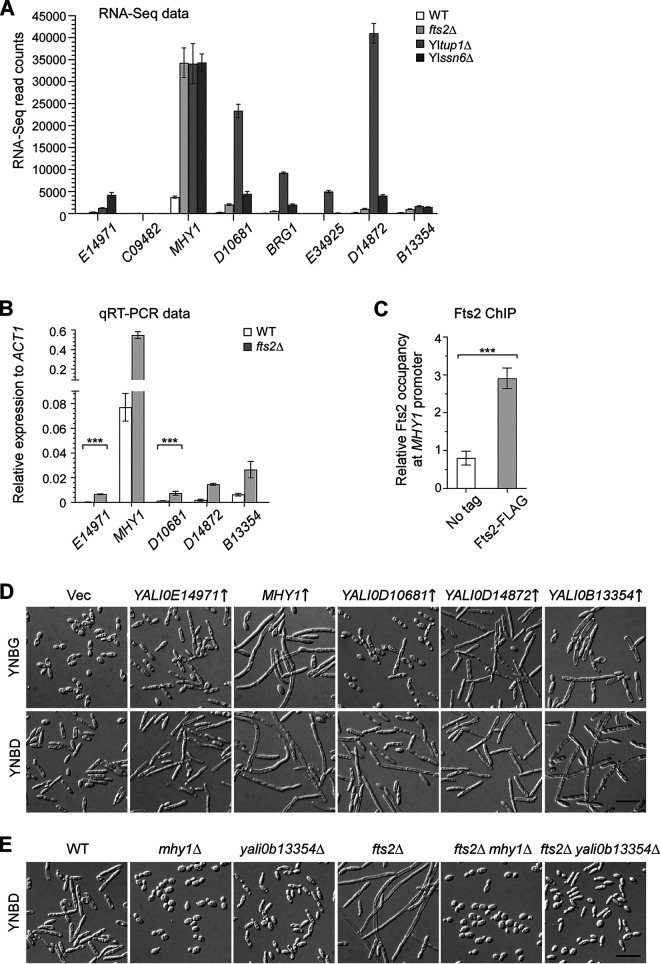
Fts2 represses a large number of transcription factor genes, some of which promote filamentation. (A) The RNA-Seq read counts of the top eight Fts2-repressed transcription factor genes in wild-type (WT), *fts2*Δ, Yl*tup1*Δ, and Yl*ssn6*Δ strains. (B) Validation of the RNA-Seq results by qRT-PCR. The transcript levels of *YALI0E14971*, *MHY1*, *YALI0D10681*, *YALI0D14872*, and *YALI0B13354* in wild-type (WT) and *fts2*Δ strains grown in YNBD medium were determined by qRT-PCR and normalized to Yl*ACT1*. Statistically significant differences are indicated by the asterisks (***, *P < *0.001). (C) Fts2 is present at the *MHY1* promoter detected by chromatin immunoprecipitation (ChIP) assay. Cells of the wild-type strain (No tag) and strain YLX524 (*FTS2-3FLAG*) carrying pINA445 vector were grown in YNBG (pH 4.0) medium at 30°C. Cells were collected at OD_600_ of ~ 0.6 for ChIP. Quantitative PCR was performed with primers at the promoter region of *MHY1*. The enrichment was presented as a ratio of *MHY1* promoter IP in Fts2-FLAG strain (bound/input) versus *MHY1* promoter IP in no-tag strain (bound/input) and was then normalized to the no-tag strain. Data were obtained from 3 independent experiments with biological duplicates in each (mean and SD). The graph shown was the ChIP result with the *MHY1* promoter region, -1069 bp to -899 bp. Statistically significant differences are indicated by the asterisks (***, *P < *0.001). (D) Five Fts2-repressed transcription factors increased filamentation in wild-type cells upon overexpression. Cells of the wild-type strain carrying pYL13 (Vec), pYL13-YALI0E14971, pYL13-MHY1, pYL13-YALI0D10681, pYL13-YALI0D14872, and pYL13-YALI0B13354 were grown in YNBG and YNBD media at 30°C. (E) The deletion of *MHY1* and *YALI0B13354* caused a drastic reduction of filamentation in wild-type and *fts2*Δ cells. Cells of the listed strains carrying pINA445 (Vec) were grown in YNBD medium at 30°C. Bars, 20 μm.

Among the 41 Fts2-repressed transcription factor genes, most of them are not known to promote filamentation except *MHY1* and *HOY1* (14, 15). *MHY1* is distinctive among these genes. *MHY1* is not only highly repressed by Fts2 (Fig. 6A and B, and Table S1), but also exhibited very high RNA-Seq read count in *fts2*Δ cells, which is the highest among transcription factor genes and very high among all Fts2-repressed genes. Hence, *MHY1* is a major target of Fts2 repression. We observed that Fts2 was present at the promoter of *MHY1* by chromatin immunoprecipitation (Fig. 6C), suggesting that Fts2 functions at the promoter of *MHY1* to repress its transcription. *MHY1* overexpression caused strong filamentation in wild-type cells (Fig. 6D), whereas *fts2*Δ *mhy1*Δ cells did not form filaments (Fig. 6E). Hence, *MHY1* is a major target of Fts2 in the repression of filamentation.

To examine whether the remaining transcription factor genes in the top 8 list may also regulate filamentation, we overexpressed 5 of them, except *YALI0C09482* and *YAL0E34925*, which exhibited the lowest RNA-Seq read counts (<80). Interestingly, overexpression of the Rme1-like transcription factor gene *YALI0E14971*, the 2 Zn(II)_2_Cys_6_ zinc cluster transcription factor genes *YALI0D10681* and *YALI0D14872*, and the bHLH transcription factor gene *YALI0B13354* all increased filamentation in wild-type cells (Fig. 6D), whereas overexpression of the GATA zinc finger transcription factor gene Yl*BRG1* (*YALI0E31757*) did not affect filamentation (data not shown). Remarkably, the deletion of *YALI0B13354* caused a drastic reduction of filamentation in wild-type cells, as well as in *fts2*Δ cells (Fig. 6E). This finding indicates that the 4 transcription factor genes *YALI0E14971*, *YALI0D10681*, *YALI0D14872*, and *YALI0B13354* promote filamentation. The repression of these genes by Fts2 may contribute to the repression of filamentation.

### Fts2 may repress the bulk of its target genes via Tup1-Ssn6.

**FIG 7:**
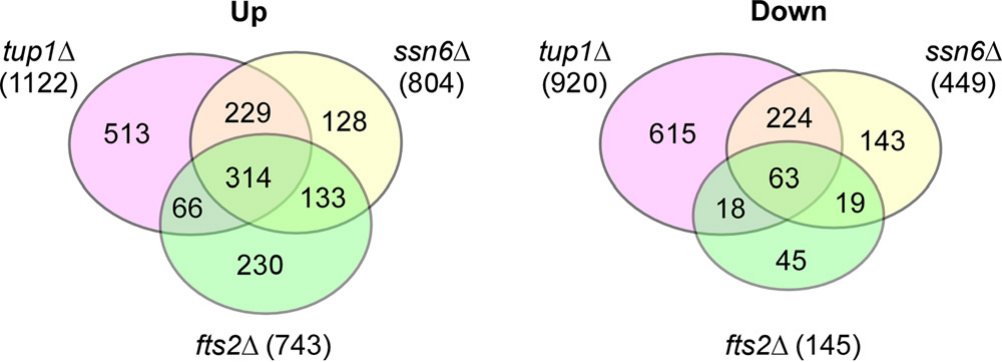
Fts2 shares a large portion of target genes with YlTup1 and YlSsn6. The Venn diagrams that represent the numbers of total, unique, and shared upregulated (Up) and downregulated (Down) genes among *fts2*Δ, Yl*tup1*Δ, and Yl*ssn6*Δ strains were shown.

To determine whether the Fts2-regulated genes may also be controlled by Tup1-Ssn6, we examined the transcript levels of Fts2-regulated genes in the RNA-Seq data sets of Yl*tup1*Δ and Yl*ssn6*Δ cells (Table S3 and Table S4). The results showed that Fts2 shares many target genes with Tup1-Ssn6 (Fig. 7). There were 1122 and 804 genes upregulated in Yl*tup1*Δ and Yl*ssn6*Δ cells, respectively. Among the 743 genes that were upregulated in *fts2*Δ cells, 380 genes (51.1%) and 447 genes (60.2%) were also upregulated in Yl*tup1*Δ and Yl*ssn6*Δ cells, respectively, whereas 314 genes (42.3%) were upregulated in both Yl*tup1*Δ and Yl*ssn6*Δ cells. Collectively, 513 genes were upregulated in either Yl*tup1*Δ or Yl*ssn6*Δ cells, indicating that 69.0% of Fts2-repressed genes were also repressed by Tup1-Ssn6. Moreover, among the 68 cell wall protein genes and 41 transcription factor genes repressed by Fts2, 66.2% (45 genes) and 90.2% (37 genes) of them were also repressed by Tup1-Ssn6, respectively (Table 1 and Table 2). In total, about two-thirds of Fts2-repressed genes are also repressed by Tup1-Ssn6. Hence, Fts2 may repress the bulk of its target genes via Tup1-Ssn6.

10.1128/msphere.00450-22.4 TABLE S3 RNA-Seq data- Yl*tup1*Δ vs WT. Download Table S3, XLSX file, 1.3 MB. Copyright © 2022 Chen et al. 2022 Chen et al. https://creativecommons.org/licenses/by/4.0/ This content is distributed under the terms of the Creative Commons Attribution 4.0 International license.

10.1128/msphere.00450-22.5 TABLE S4 RNA-Seq data- Yl*ssn6*Δ vs WT. Download Table S4, XLSX file, 1.2 MB. Copyright © 2022 Chen et al. 2022 Chen et al. https://creativecommons.org/licenses/by/4.0/ This content is distributed under the terms of the Creative Commons Attribution 4.0 International license.

There were 145 genes downregulated in *fts2*Δ cells. Of these, 100 genes (69.0%) were also downregulated in either Yl*tup1*Δ or Yl*ssn6*Δ cells (Fig. 7), indicating that the bulk of Fts2-activated genes are also activated by YlTup1 and/or YlSsn6.

### Fts2 expression is downregulated at alkaline pH and the relief of negative control by Fts2 facilitates the induction of filamentation by alkaline pH.

**FIG 8:**
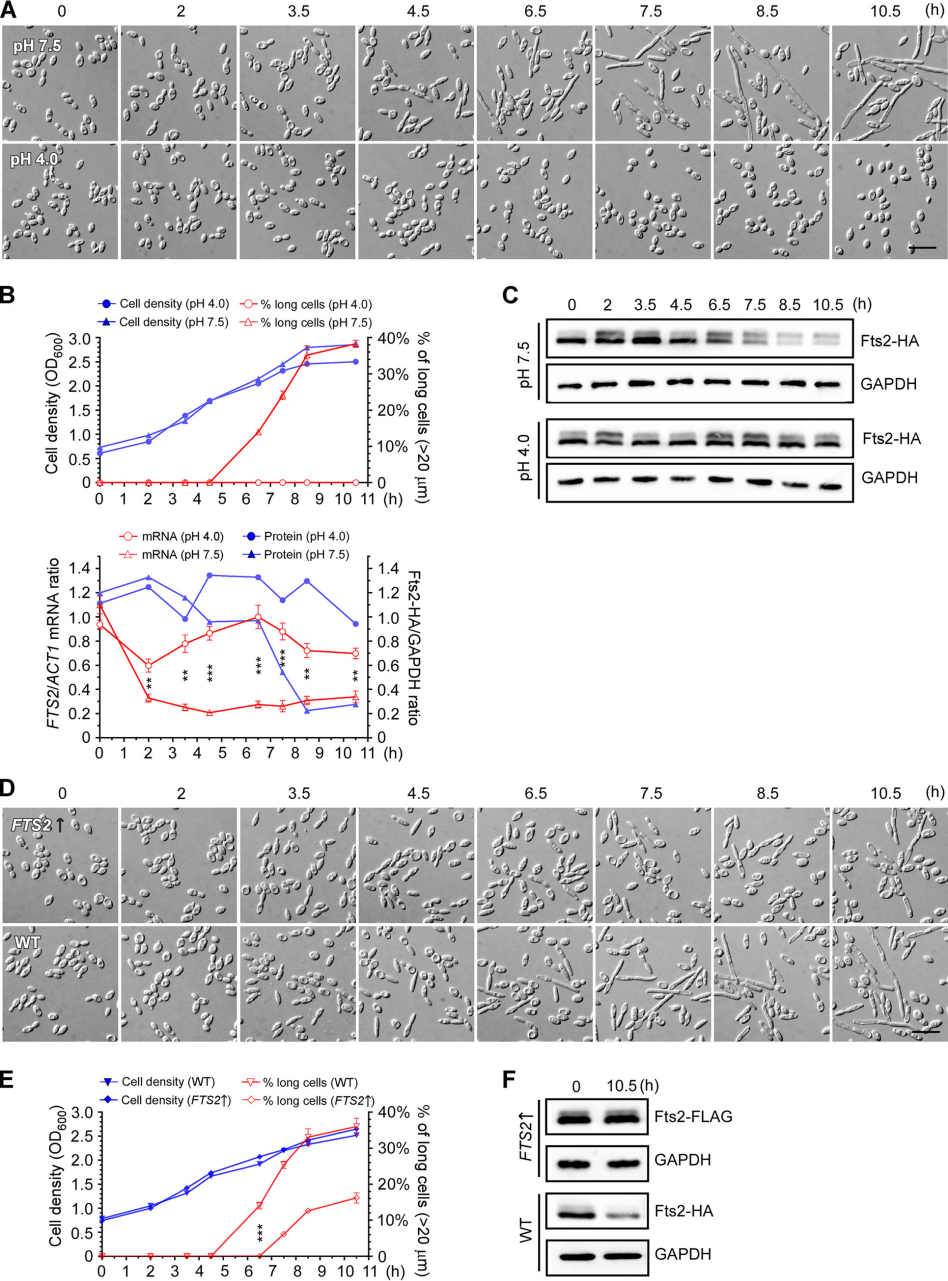
Fts2 expression is downregulated at alkaline pH and the relief of negative control by Fts2 facilitates the induction of filamentation by alkaline pH. (A) to (C) Cells of strain YLX523 (*FTS2-3HA*) carrying the pINA445 vector were grown in liquid YNBG (pH 4.0) medium to OD_600_ of ~ 0.6. Cells were collected, resuspended in fresh YNBG (pH 7.5) and YNBG (pH 4.0) media at OD_600_ of ~ 0.6, and continued to grow at 30°C. At the indicated time point, 50 mL of culture was taken out to measure OD_600_, examine cell morphology, and prepare total RNA and proteins. (A) Cell morphology at each time point. (B) *FTS2* mRNA level and Fts2 protein level decreased at alkaline pH. The mRNA level of *FTS2* at each time point was determined by qRT-PCR and normalized to Yl*ACT1* mRNA. The average of 3 independent qPCR data was shown with error bars representing SD. Statistically significant differences are indicated by the asterisks (**, *P* < 0.01; ***, *P < *0.001). The protein level of Fts2-HA was determined by immunoblotting with an anti-HA antibody. GAPDH protein was used as the control for normalization. The percentage of cells longer than 20 μm was counted. For budded cells, the mother cell and the bud that it carries were treated as one cell for the measurement of cell length. (C) The protein level of Fts2 decreased at alkaline pH. Cell lysates were prepared at each time point and subjected to immunoblotting with anti-HA and anti-GAPDH antibodies. Fts2-3HA migrated as two bands. The top band may represent the phosphorylated forms. (D) to (F) Cells of strain YLX523 carrying pYL26-FTS2-3FLAG (*FTS2*↑) or the pYL26 vector (WT) were grown in liquid YNBG (pH 4.0) medium to OD_600_ of ~ 0.6. Cells were then collected, resuspended in YNBG (pH 7.5) medium, grown, and assayed as in (A) to (C). (D) Cell morphology at each time point. Bars, 20 μm. (E) Cell density and the percentage of cells longer than 20 μm were determined. (F) The protein levels of Fts2-FLAG in the *FTS2-3FLAG* overexpression strain and Fts2-HA in the WT control strain at 0 h and 10.5 h were examined by immunoblotting.

As a repressor that prevents the yeast-to-filament transition, the transcriptional repression imposed by Fts2 is expected to be relieved during the yeast-to-filament transition. Thus, it is likely that the expression level of Fts2 might be downregulated during the induction of filamentation. To test this possibility, we examined the mRNA level and protein level of Fts2 during alkaline pH-induced filamentation. To facilitate the detection of Fts2 protein, we utilized YLX523, a wild-type strain with *FTS2* tagged C-terminally by 3 copies of HA (*FTS2-HA*) at the chromosomal locus. Cells of this strain grown at acidic pH (pH 4.0) in YNBG medium were in the oval-shaped yeast form. After being transferred to fresh YNBG medium buffered at weakly alkaline pH (pH 7.5), cells became elongated and started to filament after 4.5 h at 30^º^C (Fig. 8A, top row, and Fig. 8B, top chart). In contrast, cells were still in the yeast form during the same period after being transferred to fresh YNBG (pH 4.0) medium (Fig. 8A, bottom row). We observed that the mRNA level of *FTS2* decreased rapidly in the first 2 h after cells were transferred to pH 7.5 and remained at low level after 2 h, as detected by qRT-PCR. The steady level of *FTS2* mRNA at pH 7.5 was ~2.5-fold lower than that at pH 4.0 (Fig. 8B, bottom chart), indicating that *FTS2* transcription was downregulated in response to alkaline pH.

We also examined the protein level of Fts2-HA in the cells by immunoblotting with an anti-HA antibody (Fig. 8C). Unlike the mRNA level of *FTS2*, the protein level of Fts2 did not decrease in the first 2 h after cells were transferred to pH 7.5. However, it decreased rapidly after 6.5 h and remained at low level after 8.5 h (Fig. 8B, bottom chart, and Fig. 8C). The steady level of Fts2 protein at pH 7.5 was ~ 4-fold lower than that at pH 4.0, indicating that the protein level of Fts2 was also downregulated at pH 7.5, although it occurred later than the downregulation of *FTS2* mRNA. Together, our results demonstrate that Fts2 expression is downregulated in response to alkaline pH.

To investigate whether the downregulation of Fts2 expression at alkaline pH might be involved in the induction of filamentation by alkaline pH, we asked whether an excess of Fts2 in the cells may block filamentation. To this end, we overexpressed Fts2-FLAG on a plasmid under the control of the constitutive Yl*TDH1* (*GAPDH*) promoter in strain YLX523. This Fts2-overexpressing strain was used to repeat the alkaline-induced filamentation assay. Strain YLX523 (WT) was used as a control. We observed that alkaline-induced filamentation still occurred in cells of this strain after being transferred to YNBG medium buffered at pH 7.5. However, the onset of filamentation was delayed by about 2 h (Fig. 8D and E). Moreover, the strength of filamentation was significantly decreased, as the percentage of cells longer than 20 μm were much lower than that of the control cells (Fig. 8E). Immunoblotting revealed that the protein level of Fts2 (indicated by Fts2-FLAG) in this strain did not decrease after 10.5 h of growth at pH 7.5, whereas the protein level of Fts2-HA in the control strain did (Fig. 8F). These results indicate that an excess of Fts2 significantly impacts alkaline-induced filamentation, suggesting that the relief of transcriptional repression by Fts2 facilitates the induction of filamentation.

## DISCUSSION

The repressors of filamentation play an important role in keeping the cells in the yeast form when filamentation is not necessary. In this study, we identify Fts2, a novel repressor of filamentation in Y. lipolytica. We provide evidence that Fts2 is a transcriptional repressor and represses gene expression mainly via Tup1-Ssn6. We also show that Fts2 represses a large number of cell wall protein genes and transcription factor genes, some of which promote filamentation. Lastly, we show that Fts2 expression is downregulated in response to alkaline pH (a strong inducer of filamentation), and the relief from the negative control by Fts2 facilitates the induction of filamentation by alkaline pH. Our results provide new insights into the understanding of these transcriptional repressors and their regulation on filamentation.

We propose that Fts2 is a transcriptional repressor in the cells based on 2 observations: First, a Fts2-LexA fusion protein exhibited transcriptional repressor activity on a reporter gene. Second, transcriptomic analysis indicates that more genes are upregulated than downregulated (743 genes versus 145 genes) in *fts2*Δ cells. How does Fts2 repress gene expression? We propose that Fts2 represses gene expression mainly via the Tup1-Ssn6 corepressor. Three lines of evidence support this idea. First, Fts2-LexA fusion represses a reporter gene in a Tup1-Ssn6-dependent manner, which is similar to C. albicans CaNrg1 (25–28). Second, Fts2 interacts with YlSsn6 in co-IP assay. Third, transcriptomic analysis indicates that about two-thirds of Fts2-repressed genes are also repressed by Tup1-Ssn6. Transcriptomic analysis also suggests that Fts2 may repress about one-third of target genes in a Tup1-Ssn6-independent mechanism. Fts2 is the first transcription factor in Y. lipolytica shown to be able to repress gene expression via Tup1-Ssn6.

How does Fts2 repress filamentation? Using transcriptomic analysis, we identified the whole set of genes that are controlled by Fts2. From these, we characterized 9 cell wall protein genes and 1 transcription factor gene, *MHY1*, as the major targets of Fts2 repression. These genes were highly upregulated and exhibited very high transcript levels in *fts2*Δ cells. Of these, 7 genes except the 3 genes encoding proteins similar to S. cerevisiae Cwp1 or Tir1/Tir3 (*YALI0F07535*, *YALI0E11517*, and *YALI0E31108*) were also upregulated during alkaline-induced filamentation. Six genes including the 3 adhesin-like genes (*YALI0C11165*, *YALI0B18194*, and *YALI0C23452*), *YALI0E22286* (*CWP1*-like gene), Yl*PHR1*, and *MHY1* were highly upregulated (≥5-fold) (9). *MHY1* is a key regulator that promotes filamentation (15, 16). Yl*PHR1* is required for filamentation at alkaline pH (9). Although cells deleted for the adhesin-like gene *YALI0C23452* did not exhibit any defect in filamentation (data not shown), we observed that *YALI0C23452* increased the cell length when overexpressed together with *YALI0F19030* (9), another adhesin-like gene repressed by Fts2. The *CWP1*-like gene *YALI0E22286* is upregulated by 564-fold by alkaline pH, which is very high. *YALI0E22286* also increased the cell length when overexpressed together with *YALI0D09185* (9), another adhesin-like gene repressed by Fts2. The repression of these major targets by Fts2 might be responsible for the repression of filamentation.

In addition to the 10 major Fts2 target genes, some other Fts2-repressed cell wall protein genes and transcription factor genes might also play roles in filamentation. There are a set of 12 genes encoding proteins similar to S. cerevisiae Yps3, a plasma membrane-anchored aspartic protease involved in cell wall organization (44). Yl*MSB2* (*YALI0D00627*) encodes a protein highly similar to S. cerevisiae Msb2, a mucin that promotes filamentation (45). The 4 transcription factor genes (*YALI0E14971*, *YALI0D10681*, *YALI0D14872*, and *YALI0B13354*) promote filamentation (Fig. 6D and E). Cell polarity proteins play important roles in filamentous growth. We observed that genes encoding cell polarity proteins such as the S. cerevisiae Rho3-like GTPases Yali0B13662 and Yali0F17270, the polarisome components YlAip5 (Yali0B21626) and YlMsb3 (Yali0B22792), and the axial budding landmark component YlAxl2 (Yali0D05379) were upregulated in *fts2*Δ cells. The repression of these genes by Fts2 may collectively contribute to the repression of filamentation.

In addition to increased filamentation, *fts2*Δ cells also displayed increased resistance to peroxide, suggesting that Fts2 may also regulate the oxidative stress response. The reactive oxygen species (ROS) has been shown to promote fungal differentiation including filamentation (46–48). In C. albicans, more ROS were found to be generated in hyphal cells than in yeast-form cells (47). In S. cerevisiae, cells lacking Yno1, a NADPH oxidase-like enzyme involved in the production of ROS, displayed reduced invasive growth (48). In the RNA-Seq data sets, we observed that the genes *YALI0C20251* and *YALI0B06413* that encode oxidoreductases were highly upregulated whereas Yl*CTT1* (*YALI0E34749*), which encodes the cytosolic catalase involved in the breakdown of hydrogen peroxide, was downregulated in *fts2*Δ cells. These genes and other Fts2-regulated genes might be involved in the resistance to peroxide.

Prior to this study, the only known transcription factors that repress filamentation in Y. lipolytica are the TEA/ATTS transcription factor YlTec1 and the Zn(II)_2_Cys_6_ zinc cluster transcription factor Znc1 (Yali0B05038) (30, 31). The identification of Fts2 suggests that multiple repressors of filamentation exist in Y. lipolytica. Hence, the repression of filamentation might be rather complex. Fts2, Znc1, and YlTec1 harbor different types of DNA-binding domains. They are expected to bind to different DNA sequences, and thus may regulate different target genes. We observed that *fts2*Δ cells exhibited increased resistance to peroxide. Moreover, a large number of genes involved in nutrient transport and metabolism were upregulated and downregulated in *fts2*Δ cells. It was also reported that 161 genes and 247 genes were upregulated and downregulated, respectively, in *znc1*Δ cells during exponential growth. Most of these genes are involved in metabolism or encode proteins with catalytic, transporter, or transcription factor activity (30). Therefore, Fts2, Znc1, and YlTec1 may also have other cellular functions in addition to the repression of filamentation.

Because *fts2*Δ, *znc1*Δ, and Yl*tec1*Δ mutants all exhibited increased filamentation, Fts2, Znc1, and YlTec1 may share some common target genes. A total of 161 genes were reported to be upregulated (≥2-fold) in *znc1*Δ cells (30). Of these genes, we observed that 57 genes were also upregulated in *fts2*Δ cells, and 22 genes were also upregulated in both *fts2*Δ and Yl*tec1*Δ cells (our unpublished results). This observation indicates that Fts2, Znc1, and YlTec1 indeed share a subset of target genes. Remarkably, among the 13 genes highly upregulated (≥5-fold) in *znc1*Δ cells are 3 adhesin-like genes, *YALI0C11165*, *YALI0C08473*, and *YALI0C23452*. All 3 genes were also highly upregulated in *fts2*Δ cells, and *YALI0C23452* was also upregulated, whereas *YALI0C11165* was highly upregulated in Yl*tec1*Δ cells (our unpublished results). These results indicate that the adhesin-like genes *YALI0C11165* and *YALI0C23452* are shared targets of Fts2, Znc1, and YlTec1.

*YALI0C11165* encodes a putative GPI-anchored protein with many SSTGGADA and NGNGSDGS repeats. The Ser/Thr and Asn residues in these repeats are putative O-glycosylation and N-glycosylation sites, respectively. Yali0C11165 and its close homolog Yali0B18194, which is also highly repressed by Fts2 (Table 1 and Fig. 5C), resemble C. albicans Hyr1, a cell-surface adhesin. Its encoding gene, *HYR1*, is a filament-specific gene repressed by CaNrg1 and CaTup1 (24), two major repressors of filamentation. *YALI0C23452* encodes another putative GPI-anchored protein with many PESSEA, PKPSSEV, and PETPKPT repeats. Its encoded protein resembles the flocculin Flo11, which is a key filament-specific gene in S. cerevisiae (42, 49). Since *YALI0C11165*, *YALI0B18194*, and *YALI0C23452* are also highly upregulated during alkaline-induced filamentation (9), and the 3 encoded proteins (fused to GFP) were highly expressed in *fts2*Δ cells, we propose that *YALI0C11165*, *YALI0B18194*, and *YALI0C23452* are filament-specific genes in Y. lipolytica.

## MATERIALS AND METHODS

### Strains and media.

Y. lipolytica strains used in this study are listed in Table S5 in the supplemental material. PO1a (*MATA leu2-270 ura3-302*) was used as the wild-type strain. Culture media include yeast extract-peptone-dextrose (YPD) medium (20 g/L peptone, 10 g/L yeast extract, 2% glucose), yeast nitrogen base-dextrose (YNBD) medium (6.7 g/L yeast nitrogen base without amino acid, 1% glucose), or yeast nitrogen base-glycerol (YNBG) medium (6.7 g/L yeast nitrogen base without amino acid, 1% glycerol) supplemented with 80 mg/L of leucine, 20 mg/L of uracil, or both when required. YNBG medium was buffered to pH 4.0 or 7.5 with Na_2_HPO_4_-citric acid buffer after autoclave. The E. coli strain DH5α was used for plasmid amplification.

10.1128/msphere.00450-22.6 TABLE S5 Y. lipolytica strains used in this study. Download Table S5, PDF file, 0.7 MB. Copyright © 2022 Chen et al. 2022 Chen et al. https://creativecommons.org/licenses/by/4.0/ This content is distributed under the terms of the Creative Commons Attribution 4.0 International license.

### Microscopy.

An Olympus BX51 microscope and a Retiga 2000R CCD camera (QImaging Corporation) were used to visualize cell morphology and green fluorescent protein (GFP). The images were acquired using QCapture Suite (QImaging Corporation). For the visualization of the nucleus, yeast cells were stained with 4′,6′-diamidino-2-phenylindole (DAPI) (Sigma-Aldrich) at 1 μg/mL.

### Plasmid construction.

The plasmids used in this study are listed in Table S6. The oligonucleotides are listed in Table S7. To generate pINA445-FTS2, *FTS2* carrying the 5364-bp promoter and 517-bp 3′-UTR was amplified by PCR and inserted into *Hin*dIII-digested vector pINA445 (*CEN*, *LEU2*) using ClonExpress II One Step Cloning Kit (Vazyme Biotech Co.). To examine the localization of Fts2 in the cells, *FTS2* carrying the 5364-bp promoter was amplified by PCR and inserted into BamHI-digested vector pYL14 (pINA445 backbone, Yl*LEU2*, *GFP-T_YlURA3_*) (31), yielding pYL14-FTS2 that expresses Fts2-GFP. The *FTS2-GFP* construct in pYL14-FTS2 is functional since it restored the filamentation of *fts2*Δ cells back to the wild-type level. Similarly, to visualize the adhesin-like proteins in the cells, *YALI0C11165^1-1088^* carrying 3794-bp promoter, *YALI0B18194^1-658^* carrying 2233-bp promoter, and *YALI0C23452^1-795^* carrying 2674-bp promoter were amplified by PCR and inserted into BamHI-digested pYL14, yielding pYL14-YALI0C11165^ΔC^, pYL14-YALI0B18194^ΔC^, and pYL14-YALI0C23452^ΔC^, respectively. To overexpress the transcription factor genes including *MHY1*, the ORF of each gene plus the 500-bp 3′-UTR was amplified by PCR and inserted into pYL13 (*CEN*, *LEU2*, *P_YlTEF1_*) (31), yielding pYL13-Gene.

10.1128/msphere.00450-22.7 TABLE S6 Plasmids used in this study. Download Table S6, PDF file, 0.5 MB. Copyright © 2022 Chen et al. 2022 Chen et al. https://creativecommons.org/licenses/by/4.0/ This content is distributed under the terms of the Creative Commons Attribution 4.0 International license.

10.1128/msphere.00450-22.8 TABLE S7 Oligonucleotides used in this study. Download Table S7, PDF file, 0.6 MB. Copyright © 2022 Chen et al. 2022 Chen et al. https://creativecommons.org/licenses/by/4.0/ This content is distributed under the terms of the Creative Commons Attribution 4.0 International license.

To detect the interaction between Fts2 and YlSsn6 *in vivo*, Yl*SSN6-1×HA* carrying 2000-bp Yl*SSN6* promoter was generated by PCR amplification of Y. lipolytica genomic DNA using primer pairs 445-Ssn6-F and 445-Ssn6-HA-R. Then, it was inserted into *Hin*dIII-digested pINA445 using ClonExpress II One Step Cloning Kit, yielding pINA445-YlSSN6-HA. To generate pYL26-FTS2-3FLAG for the overexpression of Fts2-FLAG, 2 steps were employed. First, the 696-bp Yl*TDH1* glyceraldehyde-3-phosphate dehydrogenase (GAPDH) promoter (nucleotides -696 to -1 relative to the first nucleotide in the start codon) was amplified by PCR and inserted into NcoI/XbaI-digested pYL13 (31), yielding pYL26. Then, the *FTS2* ORF was tagged by *3×FLAG* by overlapping PCR and inserted into SalI-digested vector pYL26.

To generate pINA443-P_Yl_*_TEF1_*-lexA for one-hybrid assay in Y. lipolytica, the 254-bp Yl*TEF1* promoter (nucleotides −257 to −4 relative to the first nucleotide in the start codon) was amplified by PCR from genomic DNA and fused to the DNA-binding domain (a.a. 1 to 87) of the E. coli *lexA* gene by overlapping PCR. *P_YlTEF1_-lexA* was then inserted into pINA443, yielding pINA443-P_Yl_*_TEF1_*-lexA. Similarly, *P_YlTEF1_-*Yl*SSN6-lexA* and *P_YlTEF1_-FTS2-lexA* were generated and inserted into pINA443, yielding pINA443-P_YlTEF1_-YlSSN6-lexA and pINA443-P_YlTEF1_-FTS2-lexA, respectively. To generate pINA445-lexAop-P_YlACT1_-*lacZ* containing the *lacZ* gene under the control of 4 copies of the *lexA* operator and the Yl*ACT1* promoter, 575-bp Yl*ACT1* promoter (nucleotides −578 to −4 relative to the first nucleotide in the start codon) was amplified by PCR and inserted into SalI/BamHI-digested pINA445-lexAop-Yl*LEU2*-*lacZ* (31). Yl*LEU2* mini promoter was replaced by Yl*ACT1* promoter.

### Yeast strain construction.

Genes including *FTS2*, *YALI0B13354*, and *MHY1* were deleted in Y. lipolytica strains by homologous recombination. Briefly, the ~1.0-kb sequence upstream of the gene ORF (P_gene_) and the ~1.0-kb sequence downstream of the gene ORF (T_gene_) were amplified by PCR from genomic DNA, and then inserted into the flanking sites of *loxR*-Yl*URA3*-*loxP* in pYL8 (31). The resulting *P_gene_-loxR-*Yl*URA3-loxP-T_gene_* deletion cassette was used to transform yeast cells. Yeast transformants were examined by PCR to identify the ones bearing the correct gene deletion. The Yl*URA3* marker was later removed by Cre-mediated DNA recombination between *loxR* and *loxP* sites. To tag the chromosomal copy of *FTS2* with 3HA tag, *FTS2-3HA* was generated by overlapping PCR. Then, *FTS2-3HA*-*loxR-*Yl*URA3-loxP-T_FTS2_* was constructed and used to tag the chromosomal copy of *FTS2* C-terminally with 3 copies of *HA* tag by homologous recombination, yielding the yeast strain YLX523 (*FTS2-3HA:loxR/P*). Strain YLX524 (*FTS2-3FLAG:loxR/P*) was constructed similarly. The *FTS2-3HA* and *FTS2-3FLAG* fusion constructs are functional since the yeast strains displayed phenotypes similar to that of the wild-type strain in filamentation.

### Identification of *FTS2* in a mutant screen.

To look for the mutants that displayed increased filamentation, cells of the wild-type strain were transformed with the zeta-based mutagenesis cassette (MTC), zeta-*URA3*, as reported previously (50). The transformants were grown on YNBG agar. Random insertion of MTC into the chromosome created mutations. The transformants that formed fluffy or wrinkled colonies were examined for their capacity to filament in liquid medium. Those that displayed increased filamentation were analyzed by TAIL-PCR to determine the insertion site of *URA3* on the chromosome. Two insertional mutants of *yali0E23518* (*fts2*) were, thus, isolated.

### Detection of Fts2-HA and Fts2-FLAG by immunoblotting.

For the detection of the protein levels of Fts2-HA and Fts2-FLAG in the cells during alkaline-induced filamentation, cell lysates were prepared by the NaOH/TCA method. Briefly, yeast cells were collected and resuspended in 1 mL sterile water. A total of 150 μL 1.85 N NaOH/7.4% β-mercaptoethanol was added and incubated on ice for 15 min. Then, 64 μL of 100% TCA (trichloroacetic acid) was added and incubated on ice for 15 min. Cell lysates were centrifuged at high speed. The pellets were washed by 1.5 mL acetone and suspended in 2×SDS sample buffer. Standard immunoblotting procedures were used. Primary antibodies used were mouse monoclonal antibodies against HA and against GFP (Covance Research Products), and against FLAG and against GAPDH (Proteintech, Rosemont). Horseradish peroxidase-conjugated goat anti-mouse IgG (Biofly Corporation) was used as the secondary antibody.

### Co-immunoprecipitation.

Cells were collected and lysed in immunoprecipitation (IP) buffer (25 mM Tris-HCl, 1 mM EDTA, 150 mM NaCl, 5% glycerol, 1% NP-40, pH 7.4) containing protease inhibitor cocktail (Bimake Company) for 30 min on ice. The anti-FLAG affinity beads (Smart-Lifescience) were added into the lysates and incubated overnight at 4°C with rotation. After washing three times with IP buffer, the beads were resuspended into 2×SDS sample buffer, boiled, and centrifuged. Samples were analyzed by immunoblotting with the indicated antibodies.

### β-Galactosidase assay.

The β-galactosidase activity in the cells was determined by the crude cell extract assay with o-nitrophenyl-β-D-galactopyranoside (ONPG) as the substrate, as reported previously (31). Crude cell extracts were prepared by vortexing in the presence of glass beads. Protein concentration in the cell extracts was measured by the Bradford method. The specific β-galactosidase activity was normalized by the amount of total protein in each extract and was calculated according to the following formula: U = (OD_420_ × 1.7)/(0.0045 × protein concentration [mg mL^−1^] × sample volume [mL] × time [min]). The assays were performed in triplicate.

### RNA-Seq analysis.

RNA-Seq was conducted at Berry Genomics Corporation (Beijing, China) as described previously (9). Differential expression analysis between 2 conditions was performed using the DEGSeq R package (1.20.0). Differentially expressed genes were defined as those for which the adjusted *P*-value < 0.05 and the fold change ≥ 2.0.

### RNA extraction and quantitative real-time PCR analysis.

Yeast cells were grown to OD_600_ of ~ 0.6 in 50 mL cultures. Cells were collected by centrifugation at 3000 rpm for 10 min and washed twice with 10 mL sterile water. Cells were resuspended in 0.4 mL RNA isolater (Vazyme Biotech Co.), snap-frozen in liquid nitrogen, and stored at −80°C freezer. Total RNAs were extracted using RNA isolater, and purified via chloroform extraction method. The purity and yield of RNAs were examined by measuring the A_260_/A_280_ ratio using NanoDrop One Spectrometer (Thermo Scientific). RNA integrity was examined by agarose gel electrophoresis. An aliquot of 1 μg total RNA from each sample was subjected to reverse transcription using the HiScript II Q RT SuperMix for qPCR (Vazyme Biotech Co.) according to the manufacturer's protocol. Quantitative PCR (qPCR) experiments were carried out as the manufacturer's protocol suggested. The Cq values of all amplification curves were less than 28 and the melt curves of each primer set only contain a single peak. Three technical replicates data were analyzed using Bio-Rad CFX Manager software (version 3.1) with normalized mode (ΔΔCq). The unpaired two-tailed Student's T-test was used to examine the statistical significance of difference in two samples.

### Chromatin immunoprecipitation.

Yeast cells were cross-linked with 1% formaldehyde for 15 min at room temperature when OD_600_ reached about 0.6 to 0.8, and were then quenched with glycine for 5 min. Cells were collected, washed twice, and lysed in chromatin immunoprecipitation (ChIP) lysis buffer I (50 mM HEPES-KOH, 1 mM EDTA, 140 mM NaCl, 1% Triton X-100, 0.1% sodium deoxycholate, 1 mM PMSF, pH 7.5) containing protease inhibitor cocktail. After beads beating, chromatin was sonicated for 10 cycles (high output, 10 sec on, 10 sec off) using the Bioruptor (Diagenode) to produce fragments of ~300-bp length. Cell lysates was centrifuged at 16,000 × *g* for 15 min at 4°C to remove the debris. Immunoprecipitation was performed overnight at 4°C with anti-FLAG affinity beads. Beads were washed once with ChIP lysis buffer I for 10 min, once with ChIP lysis buffer II (50 mM HEPES-KOH, 1 mM EDTA, 500 mM NaCl, 1% Triton X-100, 0.1% sodium deoxycholate, 1 mM PMSF, pH 7.5) for 20 min, and once with LiCl/NP40 buffer (10 mM Tris-HCl, 1 mM EDTA, 250 mM LiCl, 0.5% NP-40, 0.5% sodium deoxycholate, pH 8.0) for 10 min. Elution was performed with 2 consecutive 10 min incubations with elution buffer (100 mM NaHCO_3_, 1% SDS) at room temperature. After reverse cross-linking and proteinase K digestion, DNA was column-purified using the Universal DNA purification kit (TIANGEN).

### Data availability.

RNA-Seq data can be found in the supplemental material.
