# Supplementary figures and images for: The Zinc Finger Transcription Factor Fts2 Represses the Yeast-to-Filament Transition in the Dimorphic Yeast Yarrowia lipolytica
Source: mSphere. 2022 Nov 21;7(6):e00450-22. doi: 10.1128/msphere.00450-22 (PMC9769893; doi:10.1128/msphere.00450-22)

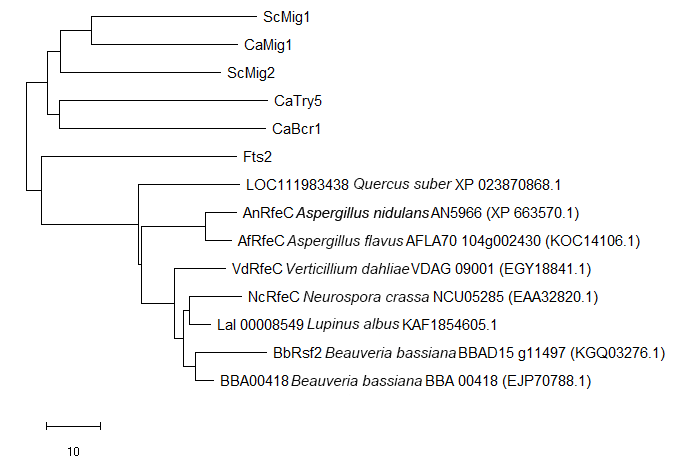

Supplement: FIG S1 [file msphere.00450-22-s0001.tif]
