# Supplementary material for: The Zinc Finger Transcription Factor Fts2 Represses the Yeast-to-Filament Transition in the Dimorphic Yeast Yarrowia lipolytica
Source: mSphere. 2022 Nov 21;7(6):e00450-22. doi: 10.1128/msphere.00450-22 (PMC9769893; doi:10.1128/msphere.00450-22)
Supplement: TABLE S2 [file msphere.00450-22-s0003.pdf]

Table S2. Functional categories of the upregulated and downregulated genes in *fts2*Δ cells.

**Function**	**Upregulated (≥2-fold)**	**Downregulated (≥2-fold)**	**Upregulated (≥5-fold)**	**Downregulated (≥5-fold)**
Nutrient transporter	114	32	22	13
Metabolism and energy	67	45	18	2
Cell wall organization and biogenesis	68	8	23	1
Transcription and transcription factor	45	2	6	0
Cell cycle	24	0	2	0
Cell polarity	15	1	2	1
Signal transduction	17	0	0	0
Stress response	11	3	3	0
RNA processing	10	0	4	0
Protease and protein degradation	11	0	4	0
Protein fate (folding, modification, maturation)	11	0	0	0
Membrane trafficking	10	0	0	0
Cytoskeleton and organization	6	0	0	0
Other cellular functions	26	7	10	0
Similar to proteins without known cellular function*	88	14	12	4
No close homologs*	220	33	71	4
**Total**	743	145	177	25

Note: * In comparison with proteins in *S. cerevisiae*, *C. albicans*, or other fungi.
