# Supplementary material for: The Zinc Finger Transcription Factor Fts2 Represses the Yeast-to-Filament Transition in the Dimorphic Yeast Yarrowia lipolytica
Source: mSphere. 2022 Nov 21;7(6):e00450-22. doi: 10.1128/msphere.00450-22 (PMC9769893; doi:10.1128/msphere.00450-22)
Supplement: TABLE S5 [file msphere.00450-22-s0006.pdf]

Table S5. ***Y. lipolytica*** **strains used in this study.**

Strain	Genotype	Source
PO1a	*MATA leu2-270 ura3-302*	1
YLX497	As PO1a except *mhy1Δ::loxR/P*	2
YLX519	As PO1a except *fts2Δ::loxR/P*	This study
YLX520	As PO1a except *yali0b13354Δ::loxR/P*	This study
YLX521	As PO1a except *fts2Δ::loxR/P mhy1Δ::loxR/P*	This study
YLX522	As PO1a except *fts2Δ::loxR/P yali0b13354Δ::loxR/P*	This study
YLX523	As PO1a except *FTS2-3HA:loxR/P*	This study
YLX524	As PO1a except *FTS2-3FLAG:loxR/P*	This study
YLX525	As PO1a except Yl*tup1Δ::loxR/P*	This study
YLX526	As PO1a except Yl*ssn6Δ::loxR/P*	This study

References

1. Barth G and Gaillardin C (1996) The dimorphic fungus *Yarrowia lipolytica*. p.313-368. In K. Wolf (ed). Non-conventional yeasts in biotechnology. Springer, Heidelberg, Germany.

2. Wu H, Shu T, Mao Y-S and Gao X-D (2020) Characterization of the promoter, downstream target genes and recognition DNA sequenced of Mhy1, a key filamentation-promoting transcription factor in the dimorphic yeast *Yarrowia lipolytica*. *Curr. Genet*. 66: 245-261.
