# Supplementary material for: The Zinc Finger Transcription Factor Fts2 Represses the Yeast-to-Filament Transition in the Dimorphic Yeast Yarrowia lipolytica
Source: mSphere. 2022 Nov 21;7(6):e00450-22. doi: 10.1128/msphere.00450-22 (PMC9769893; doi:10.1128/msphere.00450-22)
Supplement: TABLE S6 [file msphere.00450-22-s0007.pdf]

Table S6. Plasmids used in this study.

**Plasmid**	**Description**	**Source**
pINA445	pBR322 carrying *ARS68 (CEN/ARS)* and Yl*LEU2*	1
pINA445-FTS2	*FTS2* carrying 5364-bp promoter and 517-bp 3'-UTR	This study
pINA445-YlSSN6-HA	Yl*SSN6-1×HA* carrying 2000-bp promoter	This study
pYL14	pINA445 carrying *EGFP-T_{YlURA3}*	2
pYL14-FTS2	*FTS2-GFP* carrying 5364-bp *FTS2* promoter	This study
pYL14-YALI0C11165$^{\Delta C}$	*YALI0C11165$^{1\text{-}1088}$-GFP* (a.a. 1-1088) carrying 3794-bp *YALI0C11165* promoter	This study
pYL14-YALI0B18194$^{\Delta C}$	*YALI0B18194$^{1\text{-}658}$-GFP* (a.a. 1-658) carrying 2233-bp *YALI0B18194* promoter	This study
pYL14-YALI0C23452$^{\Delta C}$	*YALI0C23452$^{1\text{-}795}$-GFP* (a.a. 1-795) carrying 2674-bp *YALI0C23452* promoter	This study
pYL13	pINA445 carrying 406-bp Yl*TEF1* promoter	2
pYL13-MHY1	P_{YlTEF1}-*MHY1* plus 1185-bp 3'-UTR	3
pYL13-YALI0E14971	P_{YlTEF1}-*YALI0E14971* plus 500-bp 3'-UTR	This study
pYL13-YALI0D10681	P_{YlTEF1}-*YALI0D10681* plus 500-bp 3'-UTR	This study
pYL13-YALI0D14872	P_{YlTEF1}-*YALI0D14872* plus 500-bp 3'-UTR	This study
pYL13-YALI0B13354	P_{YlTEF1}-*YALI0B13354* plus 500-bp 3'-UTR	This study
pYL8	pBlueScript KS(+) carrying *loxR*-Yl*URA3-loxP*	2
pYL8-FTS2	P_{FTS2}-*loxR*-Yl*URA3-loxP*-T_{FTS2} in pYL8	This study
pYL8-MHY1	P_{MHY1}-*loxR*-Yl*URA3-loxP*-T_{MHY1} in pYL8	3
pYL8-YALI0B13354	$P_{YALI0B13354}$-*loxR*-Yl*URA3-loxP*-$T_{YALI0B13354}$ in pYL8	This study
pYL8-FTS2-3HA	*FTS2-3HA-loxR*-Yl*URA3-loxP*-T_{FTS2} in pYL8	This study
pRRQ2	*ARS68 (CEN/ARS),* Yl*LEU2*, *hp4d-CRE*	4
pINA443	pBR322 carrying *ARS68 (CEN/ARS)* and Yl*URA3*	Lab collection
pINA443-P_{YlTEF1}-lexA	P_{YlTEF1}-*lexA* containing 254-bp Yl*TEF1* promoter and *lexA* (a.a. 1-87)	This study
pINA443-P_{YlTEF1}-YlSSN6-lexA	P_{YlTEF1}-*YlSSN6-lexA* containing 254-bp Yl*TEF1* promoter and *lexA* (a.a. 1-87)	This study
pINA443-P_{YlTEF1}-FTS2-lexA	P_{YlTEF1}-*FTS2-lexA* containing 254-bp Yl*TEF1* promoter and *lexA* (a.a. 1-87)	This study
pINA445-lexAop-Yl*LEU2*-lacZ	*lexAop4*-P_{YlLEU2}-*lacZ* in pINA445	2
pINA445-lexAop-P_{YlACT1}-lacZ	*lexAop4*-P_{YlACT1}-*lacZ* in pINA445	This study
pYL26	pINA445 carrying the 696-bp Yl*TDH1* (GAPDH) promoter	This study
pYL26-FTS2-3FLAG	P_{YlTDH1}-*FTS2-3×FLAG*	This study

References

1. Nuttley WM, Brade AM, Gaillardin C, Eitzen GA, Glover JR, Aitchison JD and Rachubinski RA (1993)

Rapid identification and characterization of peroxisomal assembly mutants in *Yarrowia lipolytica*. *Yeast*. 9: 507–517.

2. Zhao X-F, Li M, Li Y-Q, Chen X-D and Gao X-D (2013) The TEA/ATTS transcription factor YlTec1p represses the yeast-to-hypha transition in the dimorphic yeast *Yarrowia lipolytica*. *FEMS Yeast Res*. 13: 50-61.

3. Wu H, Shu T, Mao Y-S and Gao X-D (2020) Characterization of the promoter, downstream target genes and recognition DNA sequenced of Mhy1, a key filamentation-promoting transcription factor in the dimorphic yeast *Yarrowia lipolytica*. *Curr. Genet*. 66: 245-261.

4. Richard M, Quijano RR, Bezzate S, Bordon-Pallier F and Gaillardin C (2001) Tagging morphogenetic genes by insertional mutagenesis in the yeast *Yarrowia lipolytica*. *J. Bacteriol.* 183: 3098-3107.
