# Supplementary material for: The Zinc Finger Transcription Factor Fts2 Represses the Yeast-to-Filament Transition in the Dimorphic Yeast Yarrowia lipolytica
Source: mSphere. 2022 Nov 21;7(6):e00450-22. doi: 10.1128/msphere.00450-22 (PMC9769893; doi:10.1128/msphere.00450-22)
Supplement: TABLE S7 [file msphere.00450-22-s0008.pdf]

Table S7. Oligonucleotides used in this study.

Name	Sequence (5'→3')	Use
FTS2-PF	cgctctagaactagtggatccTGCAAAGCAAGGGTGCAAGA	Amplification of P_{FTS2}
FTS2-PR	ttcctgcagcccgggggatccCTGCTCAGTTCGGTCTGCTC	Amplification of P_{FTS2}
FTS2-TF	gctatacgaagttataagcttACGGAAATACCACCGTCTCC	Amplification of T_{FTS2}
FTS2-TR	gtcgacggtatcgataagcttCGTCACGTGAACAGCAACAG	Amplification of T_{FTS2}
FTS2-5CK	ACAGAGTCCCAAATGACCGACGTC	Checking *fts2*Δ deletion
FTS2-3CK	TATGCACTTTCACTGGTACGTTGG	Checking *fts2*Δ deletion
3×HA-F	TACCCATACGATGTTC	Amplification of HA
3×HA-R	ttcctgcagcccgggggatccCTAAGCGTAGTCTGGTAC	Amplification of HA
FTS2-DQPR	GAACATCGTATGGGTACATGAGACCCTCGAGCGA CC	Overlapping PCR fusion of *FTS2* and *3×HA*
FlagL-F	GGTCGCTCGAGGGTCTCATGGACTACAAAGACCA TGACGG	Amplification of *3×FLAG*
FlagL-R	GATCCACTAGTTCTAGAGCGCTACTTGTCATCGTC ATCCT	Amplification of *3×FLAG*
FTS2-PR (into tag)	CATGAGACCCTCGAGCGACC	Overlapping PCR fusion of *FTS2* and *3×FLAG*
YALI0B13354-PF	cgctctagaactagtggatccGACCTACTACGGACCTACATA C	Amplification of $P_{YALI0B13354}$
YALI0B13354-PR	ttcctgcagcccgggggatccGACGTGTGTATGTTATGTGTC G	Amplification of $P_{YALI0B13354}$
YALI0B13354-TF	gctatacgaagttataagcttCTAGCTATCTGAGAAAGGTTTC CC	Amplification of $T_{YALI0B13354}$
YALI0B13354-TR	gtcgacggtatcgataagcttTTGCAAGTGTAGAGCTCGATT G	Amplification of $T_{YALI0B13354}$
YALI0B13354-5CK	ACGTCAGTGATCCGGTGTAG	Checking *yali0b13354*Δ deletion
YALI0B13354-3CK	CTCTTGCTCTACAGTACTTG	Checking *yali0b13354*Δ deletion
FTS2-HBF	cagcttatcatcgataagcttGCCGATACTGATGTTAACGAAT GC	Amplification of FTS2
FTS2-HBR	taaactaccgcattaaagcttTGATGCTGACCTAATCACTCAT CAG	Amplification of FTS2
YALI0C11165-GFPF	agatctagaactagtggatccCTTATTAAGCCCATGGATGAG AG	Amplification of YALI0C11165$^{1-1088}$
YALI0C11165-GFPR	accgtcgacaagcttggatccAGGGATATCAGAAGGTCCGGC AG	Amplification of YALI0C11165$^{1-1088}$
YALI0B18194-GFPF	agatctagaactagtggatccATGTACAAGTAACGGTTAATG	Amplification of YALI0B18194$^{1-658}$
YALI0B18194-GFPR	accgtcgacaagcttggatccAGCAGGGGCGGTAGCCTGGG	Amplification of YALI0B18194$^{1-658}$

YALI0C23452-GFPF	agatctagaactagtggatccGACAACCTAAATAGATAATTTGATGTG	Amplification of YALI0C23452$^{1-795}$
YALI0C23452-GFPR	accgtcgacaagcttggatccGTGCTCGGGAGTGTTACTTCCACC	Amplification of YALI0C23452$^{1-795}$
YALI0E14971-OEF	caggaattcgatatcaagcttATGAGCGAACCCTCCCCC	Amplification of YALI0E14971 ORF
YALI0E14971-OER	gtcgacggtatcgataagcttGTTATGTACATACATATTCCTGCTTC	Amplification of YALI0E14971 ORF
YALI0D10681-OEF	caggaattcgatatcaagcttATGGCCGACGCTGCCCG	Amplification of YALI0D10681 ORF
YALI0D10681-OER	gtcgacggtatcgataagcttCTTGCTATACCCACGAGATTTAAGTG	Amplification of YALI0D10681 ORF
YALI0D14872-OEF	caggaattcgatatcaagcttATGATTAGTGAGCCTTCTAGCACG	Amplification of YALI0D14872 ORF
YALI0D14872-OER	gtcgacggtatcgataagcttCGTTTCTTGGCGTGAAGAGC	Amplification of YALI0D14872 ORF
YALI0B13354-OEF	caggaattcgatatcaagcttATGGACCTCAAATCAATTGTGTTG	Amplification of YALI0B13354 ORF
YALI0B13354-OER	gtcgacggtatcgataagcttTGCTACTTGTACTTGTAACTAAC	Amplification of YALI0B13354 ORF
U1-qF	TGGTATTCTGCCGTCAACCC	qPCR for *YALI0C11165*
U1-qR	TCAGCATGACGAGCAATGGT	qPCR for *YALI0C11165*
U2-qF	ATCCTCGCTCTTGCTACCCT	qPCR for *YALI0C15004*
U2-qR	GGTGACAATGTTGGCAGCAG	qPCR for *YALI0C15004*
U22-qF	TCATCGCCTCTATCGCCAAC	qPCR for *YALI0F07535*
U22-qR	AGAGGGGAAGTTGGTCTCGT	qPCR for *YALI0F07535*
U41-qF	CTGCCCGTGTCATCTACCAG	qPCR for *YALI0E11517*
U41-qR	GCTTGTGGCACTTGTCATCC	qPCR for *YALI0E11517*
U55-qF	TACTCGTGGTGCGGAAACTC	qPCR for *YALI0D04851*
U55-qR	TCAATCTCAGTGAAGGGCCG	qPCR for *YALI0D04851*
U61-qF	TTACTGACACTGACTGCGACG	qPCR for *YALI0E22286*
U61-qR	GACGGTGACAACGGTCTTGC	qPCR for *YALI0E22286*
U69-qF	GTCGTACGTGGTTGGAGGAG	qPCR for *YALI0D09185*
U69-qR	ACGATGGACACCGTTCTCAC	qPCR for *YALI0D09185*
U107-qF	CGACACTACCACCACTTCCG	qPCR for *YALI0C23452*
U107-qR	AAGGGTCGGGAGAAGTCTGA	qPCR for *YALI0C23452*
U202-qF	AACACCACCCAGACTGTCAC	qPCR for *YALI0F19030*
U202-qR	ACGGTGACCTTGGTGGTAAC	qPCR for *YALI0F19030*
YALI0E14971-qF	TACAACGTCACACGTCCTGG	qPCR for *YALI0E14971*
YALI0E14971-qR	CCCTCAGCACTGCATGTGTA	qPCR for *YALI0E14971*
MHY1-qF	TCGAGTATGCACACGACGAG	qPCR for *MHY1*
MHY1-qR	TCGAGAGGCGAGTAAGTGGA	qPCR for *MHY1*
YALI0D10681-qF	ATGAAGTGCCATACGGGTCC	qPCR for *YALI0D10681*

YALI0D10681-qR	TTGTCGGTTCCACAGACTCG	qPCR for *YALI0D10681*
YALI0D14872-qF	TTGGTGAAGGCCGTAGACAC	qPCR for *YALI0D14872*
YALI0D14872-qR	AGCTGGTATGACTGACACGC	qPCR for *YALI0D14872*
YALI0B13354-qF	ATGAACCTGCTTCTCCCACG	qPCR for *YALI0B13354*
YALI0B13354-qR	GACAGAATGTGTGCGTGTCG	qPCR for *YALI0B13354*
FTS2-qF	TATTGCAAGTCCAAGCACGC	qPCR for *FTS2*
FTS2-qR	CTTGGAGTCCTCCTGCTTCTG	qPCR for *FTS2*
ACT1-qF	GTATGTGCAAGGCCGGTTTC	qPCR for Yl*ACT1*
ACT1-qR	GGATACCTCGCTTGGACTGG	qPCR for Yl*ACT1*
Mhy1-PF3	GTACACTGGTGGCCAAGCCC	ChIP-qPCR for the 171-bp *MHY1* promoter region (-1069 bp to -899 bp)
Mhy1-PR3	CGCTGACACCTGCCACTCCC	ChIP-qPCR for the 171-bp *MHY1* promoter region (-1069 bp to -899 bp)
445-Ssn6-F	cagcttatcatcgataagcttGTCTGAATGAACCGCCAGCCCCTCC	Generation of YlSsn6 and 1×HA fusion protein
445-Ssn6-HA-R	ccgcattaaagcttCTAAGCGTAATCTGGAACATCGTATGGGTACTTGTCCTCGGATTT	Generation of YlSsn6 and 1×HA fusion protein
N-TEF1-SF (443)	ccacacccgtcctgtggatccGGCGTAGGGTACTGCAGTCTGG	Amplification of Yl*TEF1* promoter
N-TEF1-R	TTTGAATGATTCTTATACTCAGAAGG	Amplification of Yl*TEF1* promoter
N-Ssn6-DQF (TEF1)	CCTTCTGAGTATAAGAATCATTCAAAATGACCCAACTACAAAAACTACAAACG	Overlapping PCR fusion of Yl*TEF1* promoter and Yl*SSN6*
N-Ssn6-R (for DQ LexA)	CCTGGCCGTTAACGCTTTCATCTTGTCCTCGGATTTTTCCTC	Overlapping PCR fusion of Yl*SSN6* and *lexA* (a.a. 1-87)
N-Fts2-DQF (TEF1)	CCTTCTGAGTATAAGAATCATTCAAAATGCCCCTCAAGTCGAAATCCAAG	Overlapping PCR fusion of Yl*TEF1* promoter and *FTS2*
N-Fts2-R (for DQ LexA)	CCTGGCCGTTAACGCTTTCATCATGAGACCCTCGAGCGACCAG	Overlapping PCR fusion of *FTS2* and *lexA* (a.a. 1-87)
N-LexA-F	ATGAAAGCGTTAACGGCCAGG	Amplification of *lexA* (a.a. 1-87)
LexA-R (443)	tgcgtccggcgtagaggatccCTATGGTTCACCGGCAGCC	Amplification of *lexA* (a.a. 1-87)
N-LexA-F (TEF1)	CCTTCTGAGTATAAGAATCATTCAAAATGAAAGCGTTAACGGCCAGG	Overlapping PCR fusion of Yl*TEF1* promoter and *lexA* (a.a. 1-87)
GAPDH-F (NcoI)	cgttagacgagctcagtgccatggCTGTAGGTTGGGTTGGGTGGGAGC	Amplification of Yl*TDH1* (*GAPDH*) promoter
GAPDH-R (XbaI)	ggatccactagttctagaTGTTGATGTGTGTTTAATTCAAGAATG	Amplification of Yl*TDH1* (*GAPDH*) promoter

Note: Gene sequences are written in capital letters. Restriction sites used for cloning are underlined.
